# Hypertension and renin-angiotensin system blockers are not associated with expression of angiotensin-converting enzyme 2 (ACE2) in the kidney

**DOI:** 10.1093/eurheartj/ehaa794

**Published:** 2020-10-27

**Authors:** Xiao Jiang, James M Eales, David Scannali, Alicja Nazgiewicz, Priscilla Prestes, Michelle Maier, Matthew Denniff, Xiaoguang Xu, Sushant Saluja, Eddie Cano-Gamez, Wojciech Wystrychowski, Monika Szulinska, Andrzej Antczak, Sean Byars, Damian Skrypnik, Maciej Glyda, Robert Król, Joanna Zywiec, Ewa Zukowska-Szczechowska, Louise M Burrell, Adrian S Woolf, Adam Greenstein, Pawel Bogdanski, Bernard Keavney, Andrew P Morris, Anthony Heagerty, Bryan Williams, Stephen B Harrap, Gosia Trynka, Nilesh J Samani, Tomasz J Guzik, Fadi J Charchar, Maciej Tomaszewski

**Affiliations:** Division of Cardiovascular Sciences, Faculty of Biology, Medicine and Health, University of Manchester, Manchester, UK; Division of Cardiovascular Sciences, Faculty of Biology, Medicine and Health, University of Manchester, Manchester, UK; Division of Cardiovascular Sciences, Faculty of Biology, Medicine and Health, University of Manchester, Manchester, UK; Division of Cardiovascular Sciences, Faculty of Biology, Medicine and Health, University of Manchester, Manchester, UK; School of Health and Life Sciences, Federation University Australia, Ballarat, VIC, Australia; School of Health and Life Sciences, Federation University Australia, Ballarat, VIC, Australia; Department of Cardiovascular Sciences, University of Leicester, Leicester, UK; Division of Cardiovascular Sciences, Faculty of Biology, Medicine and Health, University of Manchester, Manchester, UK; Division of Cardiovascular Sciences, Faculty of Biology, Medicine and Health, University of Manchester, Manchester, UK; Department of Cellular Genetics, Wellcome Sanger Institute, Cambridge, UK; Department of General, Vascular and Transplant Surgery, Medical University of Silesia, Katowice, Poland; Department of Treatment of Obesity, Metabolic Disorders and Clinical Dietetics, Poznan University of Medical Sciences, Poznan, Poland; Department of Urology and Uro-oncology, Karol Marcinkowski University of Medical Sciences, Poznan, Poland; Centre for Systems Genomics, School of BioSciences, The University of Melbourne, Parkville, VIC, Australia; Department of Pathology, The University of Melbourne, Parkville, VIC, Australia; Department of Treatment of Obesity, Metabolic Disorders and Clinical Dietetics, Poznan University of Medical Sciences, Poznan, Poland; Department of Transplantology and General Surgery Poznan, Collegium Medicum, Nicolaus Copernicus University, Bydgoszcz, Poland; Department of General, Vascular and Transplant Surgery, Medical University of Silesia, Katowice, Poland; Department of Internal Medicine, Diabetology and Nephrology, Medical University of Silesia, Zabrze, Poland; Department of Health Care, Silesian Medical College, Katowice, Poland; Department of Medicine and Cardiology, University of Melbourne, Melbourne, VIC, Australia; Division of Cell Matrix Biology and Regenerative Medicine, Faculty of Biology, Medicine and Health, University of Manchester, Manchester, UK; Royal Manchester Children’s Hospital and Manchester Academic Health Science Centre, Manchester University NHS Foundation Trust, Manchester, UK; Division of Cardiovascular Sciences, Faculty of Biology, Medicine and Health, University of Manchester, Manchester, UK; Division of Medicine and Manchester Academic Health Science Centre, Manchester University NHS Foundation Trust Manchester, Manchester, UK; Department of Treatment of Obesity, Metabolic Disorders and Clinical Dietetics, Poznan University of Medical Sciences, Poznan, Poland; Division of Cardiovascular Sciences, Faculty of Biology, Medicine and Health, University of Manchester, Manchester, UK; Division of Medicine and Manchester Academic Health Science Centre, Manchester University NHS Foundation Trust Manchester, Manchester, UK; Division of Musculoskeletal & Dermatological Sciences, Faculty of Medicine, Biology and Health, University of Manchester, Manchester, UK; Division of Cardiovascular Sciences, Faculty of Biology, Medicine and Health, University of Manchester, Manchester, UK; Division of Medicine and Manchester Academic Health Science Centre, Manchester University NHS Foundation Trust Manchester, Manchester, UK; Institute of Cardiovascular Sciences, University College London, London, UK; Department of Physiology, University of Melbourne, Melbourne, VIC, Australia; Department of Cellular Genetics, Wellcome Sanger Institute, Cambridge, UK; Department of Cardiovascular Sciences, University of Leicester, Leicester, UK; Leicester Biomedical Research Centre, National Institute for Health Research, Leicester, UK; Institute of Cardiovascular and Medical Sciences, College of Medical, Veterinary and Life Sciences, University of Glasgow, Glasgow, UK; Department of Internal and Agricultural Medicine, Jagiellonian University College of Medicine, Kraków, Poland; School of Health and Life Sciences, Federation University Australia, Ballarat, VIC, Australia; Department of Cardiovascular Sciences, University of Leicester, Leicester, UK; Department of Physiology, University of Melbourne, Melbourne, VIC, Australia; Division of Cardiovascular Sciences, Faculty of Biology, Medicine and Health, University of Manchester, Manchester, UK; Division of Medicine and Manchester Academic Health Science Centre, Manchester University NHS Foundation Trust Manchester, Manchester, UK

**Keywords:** ACE2, Kidney, Hypertension, Renin-angiotensin system, Antihypertensive treatment, Transcriptome, Estimated glomerular filtration rate

## Abstract

**Aims:**

Angiotensin-converting enzyme 2 (ACE2) is the cellular entry point for severe acute respiratory syndrome coronavirus (SARS-CoV-2)—the cause of coronavirus disease 2019 (COVID-19). However, the effect of renin-angiotensin system (RAS)-inhibition on ACE2 expression in human tissues of key relevance to blood pressure regulation and COVID-19 infection has not previously been reported.

**Methods and results:**

We examined how hypertension, its major metabolic co-phenotypes, and antihypertensive medications relate to ACE2 renal expression using information from up to 436 patients whose kidney transcriptomes were characterized by RNA-sequencing. We further validated some of the key observations in other human tissues and/or a controlled experimental model. Our data reveal increasing expression of ACE2 with age in both human lungs and the kidney. We show no association between renal expression of ACE2 and either hypertension or common types of RAS inhibiting drugs. We demonstrate that renal abundance of ACE2 is positively associated with a biochemical index of kidney function and show a strong enrichment for genes responsible for kidney health and disease in ACE2 co-expression analysis.

**Conclusion:**

Our results indicate that neither hypertension nor antihypertensive treatment is likely to alter the expression of the key entry receptor for SARS-CoV-2 in the human kidney. Our data further suggest that in the absence of SARS-CoV-2 infection, kidney ACE2 is most likely nephro-protective but the age-related increase in its expression within lungs and kidneys may be relevant to the risk of SARS-CoV-2 infection.

## Graphical Abstract

**Graphical Abstract:**
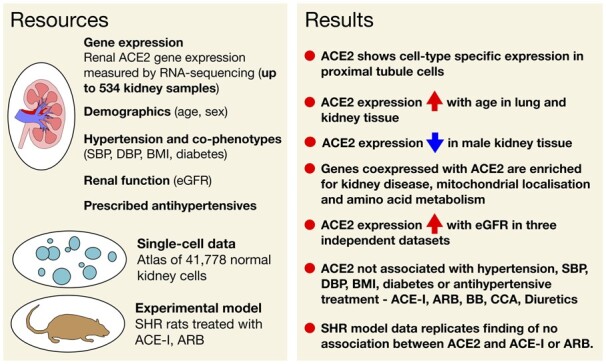

## Introduction

Severe acute respiratory syndrome coronavirus 2 (SARS-CoV-2) is responsible for the coronavirus disease 2019 (COVID-19)—the recent viral pandemic with high mortality rates and overwhelming burden on the healthcare systems globally. The virus gains entry into human host cells upon binding to angiotensin-converting enzyme 2 (ACE2)—a molecule operating both as the main trans-membrane receptor for the virus^1^ ^,^ ^2^ and a component of renin-angiotensin system (RAS)—the key blood pressure (BP) regulating cascade.^3^ ^,^ ^4^ Interestingly, elevated BP (hypertension) has been implicated as a main co-morbidity and a potential risk factor for more severe clinical outcomes of COVID-19,^5^ ^,^ ^6^ and speculation mounted that this may be due to commonly prescribed antihypertensive medications targeting RAS [i.e. angiotensin-converting enzyme inhibitors (ACE-I) or angiotensin II type 1 receptor (AT1) antagonists (ARB)]. Indeed, whilst some have hypothesized that RAS blockers enhance SARS-CoV-2 entry into the host cells^7^ ^,^ ^8^ and/or promote the organ damage in patients with COVID-19 as a result of ACE2 up-regulation, there is also evidence that RAS blockers have no influence on tissue or plasma ACE2 in animal models^9^ ^,^ ^10^ or on plasma ACE2 activity in patients.^11^ ^,^ ^12^ However, evidence for a direct effect of hypertension or RAS blockers on ACE2 expression in human tissues has remained elusive, largely because of the paucity of large gene expression datasets with matching clinical information. As the inhibition of the ACE2-SARS-CoV-2 interaction gains traction as a potential treatment strategy for COVID-19,^13^ an urgent understanding of how the key COVID-19 comorbidities alter ACE2 expression in human tissues is necessary to gain new mechanistic and therapeutic insights.

Herein, we selected the human kidney as a tissue of key importance to BP regulation, RAS and COVID-19 and examined how hypertension (as well as its major metabolic co-phenotypes) related to ACE2 expression.^7^ ^,^ ^14–17^ We further explored if RAS blockers were associated with changes in ACE2 expression taking advantage of information on antihypertensive treatment in patients whose kidney transcriptomes were characterized by RNA-sequencing. Finally, we validated some of the key observations in other human tissues and a controlled experimental model using kidneys from spontaneously hypertensive rats (SHRs) treated with ACE-I and ARB.

## Condensed methods

Our discovery dataset consisted of up to 436 human kidney samples collected by the *moleculAr analysis of human kiDney-Manchester renal tIssue pRojEct* (ADMIRE), the TRANScriptome of renaL human TissuE Study (TRANSLATE),^18–22^ and its extension (TRANSLATE-T, ‘zero time’ pre-implantation biopsy prior to transplantation),^20^  *moleculaR analysis of mEchanisms regulating gene exPression in post-ischAemic Injury to Renal allograft* (REPAIR) and *Renal gEne expreSsion and PredispOsition to cardiovascular and kidNey Disease* (RESPOND) studies. The replication was based on 98 ‘normal control’ kidneys from the Cancer Genome Atlas (TCGA, https://www.cancer.gov/tcga). Human kidney transcriptome profiling in all studies was conducted by RNA-sequencing.^20–22^ Gene expression was quantified in transcripts per million and normalized using logarithmic transformation, quantile normalization and rank-based inverse normal transformation, as reported before.^20^

Analyses of association between ACE2 expression and hypertension as well as other demographic and clinical phenotypes [i.e. age, sex, body mass index (BMI), diabetes, clinic BP, estimated glomerular filtration rate (eGFR)] were conducted using ordinary least square linear regression model adjusted for age, sex (as appropriate), other demographic/clinical variables, top three genetic principal components (PCs) and surrogate variables (SVs) in both discovery and replication (if available) datasets. The SVs were built for each model separately and the optimized number was determined by ‘sva’ package^23^ in R. The analysis of the association between renal expression of ACE2 and each of the antihypertensive classes (ACE-I, ARB, beta-blockers, calcium channel antagonists, diuretics, or others) was adjusted for age, sex, BMI, diabetes, top three genetic PCs and SVs, and further corrected for the inter-correlation between the drug classes (using ‘gee’ R package).

The co-expression analysis stratified on renal expression of ACE2 was conducted using multivariate regression and adjusted for age, sex, diabetes, hypertension, three genetic PCs, and ‘sva’ determined SVs in both the discovery and replication dataset separately. A kidney gene was considered as co-expressed with ACE2 if the direction of its association with ACE2 was consistent in the discovery and replication datasets and the corresponding level of statistical significance survived a correction for multiple testing in both.

Replication of association between kidney ACE2 expression and eGFR was examined in a total of 315 kidney samples from five renal gene expression datasets curated by *Nephroseq* (www.nephroseq.org) and an additional ‘look-up’ in an independent dataset of 95 human kidneys.^24^

The analysis of association between ACE2 expression and both age and sex in non-renal tissues of relevance to hypertension/BP regulation was conducted using Genotype-Tissue Expression (GTEx) project.^25^ The multivariate regression models were adjusted for age, sex (as appropriate), GTEx-specific variables, top three genetic PCs, a tissue-specific number of SVs using the ‘Limma’ R package.

Mapping of ACE2 on specific cell-types was conducted using single-cell RNA-sequencing data for the human adult kidney.^26^

The analysis of association between renal ACE2 expression in response to losartan and perindopril, we conducted using inbred male SHRs from the Animal Resources Centre (Canning Vale, Western Australia). At 10 weeks old, SHR were implanted with minipumps (Alzet model 2004, Durect Corp Cupertino, CA, USA) for infusions of either the vehicle (normal saline), losartan (7.5 mg/kg/day), or perindopril (1 mg/kg/day). Spontaneously hypertensive rats received infusions for 4 weeks and were sacrificed according to the standard protocols. Angiotensin-converting enzyme 2 expression in the kidney was quantified using quantitative real-time PCR.

The statistical significance of all analyses was computed with an adjustment for multiple testing.

Detailed information on materials and methods is available in the Supplementary material online.

## Results

### Renal expression of angiotensin-converting enzyme 2—insights from kidney transcriptome profiling, co-expression analysis, and single-cell experiments

**Figure 1:**
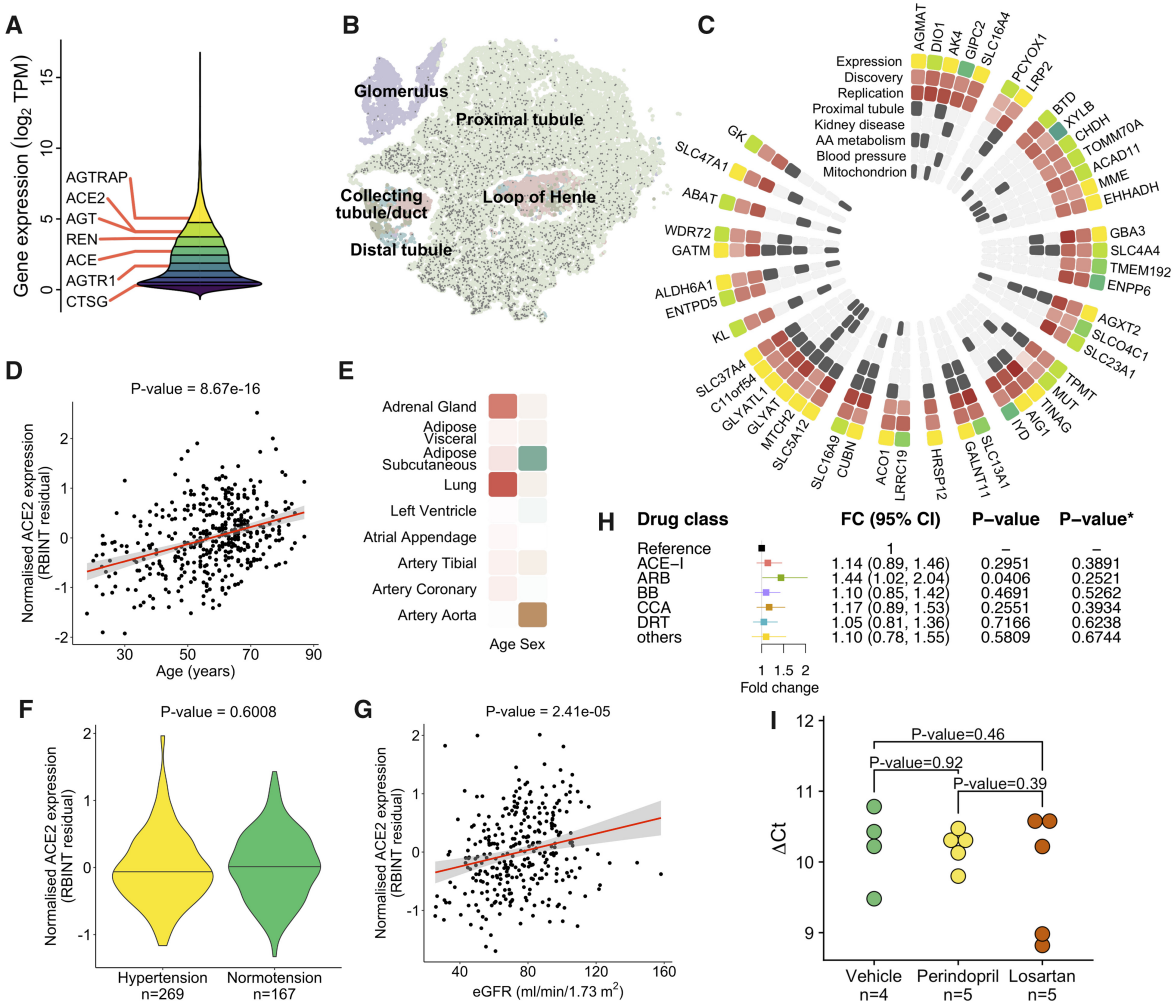
(*A*) The distribution of gene expression values in the human kidney transcriptome derived from 436 samples of the discovery population. Deciles of the distribution are shown as coloured regions from least expressed decile (dark purple) to most highly expressed (yellow). Renal expression of renin-angiotensin system genes is labelled (ACE, angiotensin I converting enzyme; ACE2, angiotensin I converting enzyme 2; AGT, angiotensinogen; AGTR1, angiotensin II receptor type 1; AGTRAP, angiotensin II receptor-associated protein; CTSG, cathepsin G; REN, renin). Expression units are transformed (log2 of the sum of transcripts per million plus a constant offset of 1). (*B*) Two-dimensional t-SNE representation of cells from normal kidney tissue. Angiotensin-converting enzyme 2 expressing cells are marked with a dark grey circle. Cells are coloured by their location in the nephron segment. t-SNE, t-distributed stochastic neighbour embedding. (*C*) Heatmap of 47 renal genes co-expressed with angiotensin-converting enzyme 2, genes are grouped and ordered by chromosomal location starting at the top and proceeding clockwise. Expression—quantile of expression in the distribution of the kidney transcriptome, colouring consistent with that in (*A*). Discovery—direction and strength of statistical association (t-statistic) in the discovery resource, positive association is shown from white (least strongly associated) to dark red (most strongly associated). Replication—direction and strength of statistical association (t-statistic) in the replication resource, colouring identical to ‘Discovery’. Proximal tubule—overlaps with the set of computational cell-type markers for proximal tubule cells. Kidney disease—overlaps with ‘renal disease’ set from genetic association database. AA-metabolism—present in REACTOME ‘Metabolism of amino acids and derivatives’. Blood pressure—present in the manually curated list of blood pressure genetically associated genes. Mitochondrion—annotated with ‘GO:0005739’ gene ontology term. (*D*) Association between renal expression of angiotensin-converting enzyme 2 and age in the discovery dataset. RBINT—the residual of normalized angiotensin-converting enzyme 2 expression, *P*-value—level of statistical significance. (*E*) Heatmap of association between angiotensin-converting enzyme 2 expression and age and sex in the selected human tissues from Genotype-Tissue Expression project. The degree of positive association with age is coloured from white to dark red. A significant association with female sex is shown as green and with male sex as brown, less significant results scale towards white. (*F*) Difference in renal expression of angiotensin-converting enzyme 2 between hypertensive and normotensive individuals in the discovery dataset, *n*—number of individuals. (*G*) Association between renal expression of angiotensin-converting enzyme 2 and estimated glomerular filtration rate (eGFR) in the discovery dataset. (*H*) The association between renal expression of angiotensin-converting enzyme 2 and each individual antihypertensive drug class, reference—hypertensive patients not on antihypertensive treatment, FC—fold change, 95% CI—95% confidence interval, *P*-value—nominal level of statistical significance from the generalized estimation equation (GEE) model, *P*-value*—level of statistical significance after adjustment for multiple testing. T vs. N, treatment vs. no treatment. (*I*) Renal angiotensin-converting enzyme 2 expression after 4-week treatment with vehicle (normal saline), perindopril or losartan in spontaneously hypertensive rats (SHR). Data are ΔCt values from quantitative real-time PCR, *P*-value, level of statistical significance for a difference in ΔCt values from *t*-test; *n*, number of animals in each group.

We first processed RNA-sequencing-derived gene expression profiles of 436 kidneys from our discovery resource (Supplementary material online, *Table S1*) and uncovered 21 203 kidney genes. The key RAS genes showed from strong to moderate expression in the kidney with ACE2 in the top 20% of the renal gene expression signal distribution (*Figure 1A*). The analysis of human kidney single-cell data^26^ revealed a cell-type-specific pattern of ACE2 expression, with proximal tubule cells showing the strongest expression signal (*Figure 1B*). Our co-expression analysis conducted in both the discovery and replication datasets uncovered 47 kidney genes tightly correlated with ACE2 (*Figure 1C)*. Twenty-three (49%) of these genes showed expression specific to proximal tubule at the single-cell level (*Figure 1C*). The ACE2 co-expressed genes were enriched for amino acid metabolism (*P* = 1.89 × 10^−5^, *Figure 1C*), mitochondria (*P* = 1.92 × 10^−17^, *Figure 1C*), kidney disease (*P* = 1.88 × 10^−10^, *Figure 1C*), and weakly for BP regulation (*P* = 0.0225, *Figure 1C*). While ACE was not within the top renal genes correlated with ACE2, it showed nominally significant positive association with ACE2 in both the discovery resource (*P* = 0.0327) and TCGA (*P* = 0.0032), consistent with previous studies.^27^ Taken together, these data show that ACE2 is a highly abundant kidney gene with a cell-type-specific pattern of expression in the proximal tubule and functional overlap with metabolic processes of key relevance to human health and disease.

### The effect of sex and age on angiotensin-converting enzyme 2 expression in the kidney and other human tissues

Angiotensin-converting enzyme 2 is encoded by a gene on the short arm of the X chromosome (Xp22.2) and was reported to escape X chromosome inactivation (XCI) in some human tissues.^28^ To examine whether there is the XCI-driven sex bias in the renal expression of ACE2, we compared male and female gene expression profiles from 436 kidneys within the discovery dataset and 98 renal tissue samples from the replication resource. We first confirmed that the sex-specific X-chromosomal and Y-chromosomal gene controls (XIST and RPS4Y1, respectively) showed the expected sex-specific pattern of expression in kidneys from both gene expression datasets (Supplementary material online, *Figure S1A*). Our discovery analysis showed that women have ∼1.36-fold higher expression of ACE2 in the kidney when compared with men (*P* = 8.6 × 10^−9^) (Supplementary material online, *Figure S1B*). We then replicated this observation in an independent population—TCGA (*P* = 8.86 × 10^−5^) (Supplementary material online, *Figure S1C*). In the combined analysis of 534 samples, women had ∼1.4-fold higher level of renal ACE2 expression when compared with men (Supplementary material online, *Figure S1D*). We then examined how sex influences ACE2 expression in the lung and several other relevant tissues from the GTEx project.^25^ In some tissues (i.e. subcutaneous adipose tissue), ACE2 showed higher expression in women than men, while in others (i.e. aorta), there was the opposite pattern of sex-specificity in ACE2 expression (*Figure 1E*).

Age showed a positive association with the renal expression of ACE2 in the discovery dataset (8.67 × 10^−16^) (*Figure 1D*). The directionality of the age-renal ACE2 relationship was consistent in the replication resource but the association did not reach the level of statistical significance (*P* = 0.2540), possibly because of the smaller number of samples in TCGA. Similar to the findings in the kidney, we detected a statistically significant positive association between age and the expression of ACE2 in lungs from the GTEx (*P* = 1.62 × 10^−4^, Supplementary material online, *Figure S1E*). In the majority of the examined human tissues, the direction of the association between age and ACE2 expression was consistent with that observed in the kidney and the lungs (*Figure 1E*).

Collectively, these data show that ACE2 exhibits a heterogeneous tissue-dependent sex-specific expression pattern in human tissues and that expression of ACE2 tends to increase with age in the kidney, lungs and the majority of tissues of relevance to cardiovascular system.

### Analysis of association between renal expression of angiotensin-converting enzyme 2, hypertension, and other clinical phenotypes of potential relevance to coronavirus disease 2019

Taking advantage of clinical information available in our discovery resource, we examined whether hypertension and other comorbidities/phenotypes of potential relevance to COVID-19 (including diabetes, BMI, eGFR) were associated with renal expression of ACE2. Our analysis, conducted in 269 hypertensives and 167 normotensives, revealed no association between human hypertension and ACE2 expression in the kidney (*P* = 0.6008) (*Figure 1F*, Supplementary material online, *Table S2*). An additional sensitivity analysis restricted to individuals who were not on antihypertensive treatment confirmed no association between ACE2 and hypertension (Supplementary material online, *Figure S1F* and *Table S2*) as well as clinic systolic BP (*P* = 0.358) and diastolic BP (*P* = 0.303). Neither diabetes nor BMI showed an association with kidney expression of ACE2 (*P* = 0.8445 and *P* = 0.8843, respectively) (Supplementary material online, *Figure S1G* and *Table S2*). However, we detected a significant positive association between renal expression of ACE2 and eGFR (*P* = 2.41 × 10^−5^) (*Figure 1G*, Supplementary material online, *Table S2*). We then replicated this finding in two additional studies. Firstly, we conducted a meta-analysis of correlation between tubular ACE2 expression and eGFR in 315 renal transcriptomes from an independent resource—*Nephroseq* (*P* = 1.19 × 10^−17^) (Supplementary material online, *Figure S1I* and *Table S3*, www.nephroseq.org). Secondly, our look-up in a gene expression dataset from 95 renal tubule samples^24^ confirmed the directionally consistent association between ACE2 and eGFR (*P* = 0.0013). Taken together, these data show that renal expression of ACE2 is positively associated with a biochemical index of kidney function but not with hypertension or other cardiovascular/metabolic comorbidities.

### The effect of antihypertensive medications on the renal expression of angiotensin-converting enzyme 2—analysis of human and rat kidneys

Of 269 hypertensive individuals in our discovery tissue resource, 221 were on antihypertensive treatment. We first confirmed that antihypertensive therapy (as a simple binarized variable) was not associated with expression of ACE2 in the kidney (*P* = 0.4176). We then allocated each prescribed antihypertensive medication into one of six categories consistent with the six main BP-lowering drug classes, in 160 individuals with detailed information on their pharmacological therapy (Supplementary material online, *Figure S1H*). After replacing the binarized antihypertensive treatment indicator with the variables indicative of the six classes and after correction for inter-correlation between the drug classes, we re-examined if renal ACE2 expression was associated with individual antihypertensive classes. There was a nominally significant association with ARB but no significant associations after the adjustment for multiple testing (*Figure 1H*).

In the absence of independent datasets with RNA-sequencing profiles of human kidneys with matching information on antihypertensive treatment, we used the kidneys from SHRs treated for 4 weeks with an ACE-I and an ARB. These experiments in essence corroborated the observations from the human kidneys—there was no statistically significant difference in renal ACE2 expression between the treatment arms (*Figure 1I*). Collectively, these data show that there is no association between commonly prescribed antihypertensive medications and renal expression of ACE2.

## Discussion

Despite widespread speculation that hypertension (and especially drugs that inhibit RAS) would be associated with increased expression of ACE2 and that this in turn, lead to increased susceptibility to COVID-19, our study revealed no effect of either on the renal expression of ACE2. We also showed that while age and sex changed the abundance of ACE2 in human tissues, common metabolic comorbidities of hypertension, including diabetes and obesity index were not associated with renal expression of ACE2. Finally, we revealed enrichment for amino acid metabolism, mitochondria, and kidney disease in the renal ACE2 co-expression analysis and demonstrated a positive association between renal ACE2 expression and a biochemical index of kidney function in the absence of the SARS-CoV-2 infection.

It is widely acknowledged that while viral pneumonitis is the main clinical manifestation of SARS-CoV-2 infection, COVID-19 is a multi-organ disease^29^ affecting the cardiovascular system, renal-urinary tract, and other organs and tissues. Recent studies have revealed the tropism of SARS-CoV-2 to the renal epithelium^17^ as well as endothelium^13^ ^,^ ^30^ and post-mortem electron microscopy has demonstrated the presence of viral inclusion structures within the kidney.^13^ ^,^ ^29^ ^,^ ^31^ The autopsy findings of patients who died of COVID-19 confirmed the prominent structural damage of tubular renal epithelium^32^ and it appears that the viral invasion of the kidney parenchyma may (at least to some extent) contribute to acute kidney injury in patients with COVID-19.^33^ Importantly, ACE2—a key trans-membrane receptor used by SARS-CoV-2 to gain entry to the host cells^2^ ^,^ ^13^ ^,^ ^34^—is very abundant in the human kidney,^35^ ^,^ ^36^ possibly more so than in the lungs.^33^ These observations suggest that the kidney is one of the key target tissues for SARS-CoV-2.

Our kidney transcriptome profiling studies mapped a strong cell-specific ACE2 expression signal to the proximal tubule and showed a strong enrichment for this cell-type amongst the genes showing the highest level of renal co-expression with ACE2. The over-representation of genes that encode different enzymes and molecular transporters uncovered by our co-expression analysis is consistent with previously reported, but not widely acknowledged, ACE2’s activity as a carboxypeptidase and its role in metabolic regulation, i.e. acting as a chaperone for amino acid transport and metabolism.^37–39^ We also observed strong enrichment for gene-products that localize in the mitochondria—this lends support to the hypothesis that viral disruption of mitochondrial oxygen sensing mechanisms may be relevant to SARS-CoV-2-driven hypoxia.^40^ In addition, we reveal that many ACE2 co-expressed kidney genes [i.e. LRP2 (megalin), CUBN (cubilin), GALNT11 (polypeptide N-Acetylgalactosaminyltransferase 11)] have a well-established role in renal physiology, i.e. reabsorption processes in the proximal tubule and regulation of urinary excretion of protein.^41^ ^,^ ^42^ Most importantly, our analyses conducted across three independent datasets of human renal tissue (totalling 720 individuals) provide strong and consistent evidence for a positive association between renal expression of ACE2 and eGFR. Collectively, these data indicate that ACE2 is positively correlated with a measure of kidney function and are consistent with previous observations on i.e. reduction in renal content of ACE2 in overt kidney disease.^43^

Previous studies conducted on various tissues from experimental models and a few studies using human blood reported inconsistent associations between ACE2 abundance and BP/hypertension.^44^ Using the largest available dataset of human kidneys with matching clinical information, we detected no association between renal expression of ACE2 and human hypertension or clinic BP. We also observed only a very weak enrichment for BP regulation in our kidney ACE2 co-expression analysis. Thus, the renal expression of ACE2 is unlikely related to the reported over-representation of patients with elevated BP amongst those with COVID-19. Indeed, while almost half of Chinese^6^ and Italian^5^ patients diagnosed with COVID-19 were hypertensive and hypertension was more prevalent amongst those who died at intensive care units than in those who were discharged^5^ or those with more severe than non-severe COVID-19,^6^ these associations were not adjusted for age—a key correlate of COVID-19-related morbidity and mortality. Being older, male sex, higher BMI, and diabetes have been linked to a higher risk of infection and/or adverse outcomes in patients with COVID-19.^6^ Of these factors, only sex and age showed an association with renal ACE2 expression in our analyses. The effect of sex on ACE2 expression is strongly dependent on the type of tissue and suggests that ACE2 escapes XCI inactivation only in selected human tissues (i.e. in the kidney) possibly as a result of more subtle tissue-specific regulatory mechanisms.^28^ The relevance of this heterogeneous sex-specific tissue expression in relevance to SARS-CoV-2 requires further study. The age-related increase in ACE2 was apparent in both the kidney and the lungs and directionally consistent in the other examined human tissues as well as a recent study using nasal epithelium.^45^ Whether this age-driven increase in ACE2 represents a ubiquitous molecular mechanism enhancing the viral entry into the host cells or protecting them from a virus-mediated organ injury (through, e.g. increased degradation of angiotensin II and/or synthesis of angiotensin 1-7) ^1^ ^,^ ^8^ ^,^ ^13^ ^,^ ^33^ ^,^ ^46^ ^,^ ^47^ remains to be established.

Our study provides an important new insight into how pharmacological blockade of RAS (a well-established strategy in the management of hypertension and other cardiovascular diseases) relates to ACE2 expression and its contemplated role in COVID-19-driven organ injury. Indeed, the RAS inhibitors (through their potential effects on ACE2 expression) have been considered as both potentially protective and harmful to patients infected by SARS-CoV-2. While ACE2 is not a direct molecular target of the RAS blockers, ACE-I reduces the levels of the ACE2 substrate (angiotensin II) while ARB block the angiotensin II-AT1 receptors interaction and thus have been proposed to attenuate the angiotensin II-mediated organ injury.^46–50^ On the other hand, RAS blockers have been hypothesized to enhance the entry of SARS-CoV-2 into host cells and enhance the severity of organ damage in patients with COVID-19, possibly as a result of up-regulated ACE2 expression.^7^ ^,^ ^8^ ^,^ ^48^ There is a widely acknowledged paucity of data linking ACE-I and ARB with ACE2 expression in the human tissues of relevance to COVID-19.^46^ Indeed, the available evidence on how RAS blockers relate to ACE2 expression is based mostly on experimental models^47^ ^,^ ^51^ ^,^ ^52^ and clinical studies conducted using blood.^53–55^ While some of the experimental studies^47^ ^,^ ^51^ ^,^ ^52^ found an association between ACE2 and RAS blockers, previous human studies reported that circulating levels of ACE2 were not related to these classes of antihypertensive medications.^54^ ^,^ ^55^ The most recent analysis of individuals with heart failure concluded that ACE inhibitors and ARBs were not associated with increased plasma concentrations of ACE2.^53^ While ACE2 operates mostly as a tissue enzyme^46^ ^,^ ^56^ ^,^ ^57^ and there is no evidence that blood levels of ACE2 correlate positively with its expression in the kidney, the results of the previous clinical studies on blood^53–55^ are largely consistent with our observations. The demonstrated lack of association between RAS blockers and renal expression of ACE2 shown in our study makes it unlikely that antihypertensive treatment drives kidney up-regulation of the key functional receptor for SARS-CoV-2—the cause of COVID-19. These observations are in line with the vast majority of observational studies and trials—with a recent living meta-analysis concluding that use of RAS blockers is not associated with worse outcomes of COVID-19 disease or higher risk of SARS-CoV-2 test positivity in symptomatic individuals.^58^ Currently, it remains uncertain whether ACE-I and ARBs use is associated with the risk of mild or asymptomatic disease or improves COVID-19 outcomes.^58^ To this end, our data also lend support to the clinical consensus (endorsed by International Society of Hypertension, European Society of Hypertension and European Society of Cardiology) not to discontinue RAS blocker in patients with COVID-19.^49^ ^,^ ^59^

Our study is based on a retrospective analysis. We also acknowledge that the discovery kidney resource brings several renal tissue collections together; this is necessary to maximize the sample size and optimize the power of the study. While samples were collected at different sites for the purpose of this project, we used consistent standards/operating procedures of tissue collection, processing, and phenotyping and all the gene expression data were processed and normalized using one centralized computational pipeline. Finally, the breadth of clinical information available for patients whose kidney samples were processed by RNA-sequencing may be not as extensive as that for some studies collecting more accessible types of biological materials (i.e. blood or urine), and therefore we could not include all potential clinical cofounders (i.e. heart failure) directly in our statistical models or in the sensitivity analyses. However, we have applied a very careful mitigation strategy to reduce the potential influence of measured and latent cofounding (of both technical and biological origin) on the results of our gene expression analysis. Furthermore, our findings are based on the largest available discovery dataset with >400 human kidneys and wherever feasible—replicated in independent resources. We also provide data from other human organs as context to our findings and make use not only of whole tissue transcriptome profiling but also—single-cell investigations and the experimental model of human hypertension (SHR). The latter provides a unique controlled and genetically uniform model to study a gene expression without potential interference of genetic heterogeneity associated i.e. with response to drug treatment. We appreciate though that given a limitation pertaining to the number of animals in these studies, they should be interpreted as preliminary and warranting a confirmation in further experimental models.

In summary, we reveal findings of potential biological and epidemiological importance to the SARS-CoV-2 infection, i.e. the signature of age of ACE2 expression in tissues of relevance to COVID-19 and the lack of association between common cardiovascular and metabolic comorbidities of COVID-19 (i.e. hypertension, diabetes) on the renal expression of the SARS-CoV-2 entry receptor. Furthermore, the lack of association between renal expression of ACE2 and RAS blockers demonstrated in this study provides an important molecular argument in favour of safety of commonly prescribed BP-lowering medications (RAS blockers) in patients with COVID-19. Finally, our data suggest that in the absence of SARS-CoV-2, renal ACE2 is positively associated with eGFR and lends support to the notion that renal ACE2 plays a more significant role in the local control of proximal tubule metabolism and kidney function rather than in systemic BP regulation.^37^

## Data availability

The data supporting the findings from these investigations are availbale within the article and the supplementary material or are available upon reasonable request to the authors.

## Supplementary material

Supplementary material is available at *European Heart Journal* online.

## Funding

This work was supported by British Heart Foundation project grants [PG/17/35/33001 and PG/19/16/34270] and Kidney Research UK [grant RP_017_20180302] to M.T., British Heart Foundation Personal Chair [CH/13/2/30154] and Manchester Academic Health Science Centre: Tissue Bank Grant to BK, Medical University of Silesia [grants KNW-1-152/N/7/K to J.Z. and KNW-1-171/N/6/K to W.W.]. F.C. and S.H. were supported by a National Health and Medical Research project grant [APP1104686]. T.J.G. was supported by the European Research Council [InflammaTENSION; ERC-CoG-726318], T.J.G. also acknowledges support from ERA-CVD [PLAQUEFIGHT/5/2018]. L.M.B. acknowledges support from National Health and Medical Research Council Program [Grant APP1055214] and Medical Research Future Fund [APP 1175865]. G.T. is supported by Open Targets and the Wellcome Trust [grant WT206194]. E.C.G. is supported by the Gates Cambridge Scholarship [OPP1144]. Access to TCGA kidneys and GTEx data has been granted by NIH [approvals 50804-2 and 50805-2]. The results published here are in part based upon data generated by the TCGA Research Network: https://www.cancer.gov/tcga. We thank the Oxford Genomics Centre at the Wellcome Centre for Human Genetics funded by Wellcome Trust [grant reference 203141/Z/16/Z] for the generation and initial processing of sequencing data.

**Conflict of interest:** none declared.

## Supplementary Material

ehaa794_Supplementary_Data

## References

[1] Kuba K, Imai Y, Rao S, Gao H, Guo F, Guan B, Huan Y, Yang P, Zhang Y, Deng W, Bao L, Zhang B, Liu G, Wang Z, Chappell M, Liu Y, Zheng D, Leibbrandt A, Wada T, Slutsky AS, Liu D, Qin C, Jiang C, Penninger JM. A crucial role of angiotensin converting enzyme 2 (ACE2) in SARS coronavirus-induced lung injury. Nat Med 2005;11:875–879. 16007097 10.1038/nm1267 PMC7095783

[2] Hoffmann M, Kleine-Weber H, Schroeder S, Krüger N, Herrler T, Erichsen S, Schiergens TS, Herrler G, Wu NH, Nitsche A, Müller MA, Drosten C, Pöhlmann S. SARS-CoV-2 cell entry depends on ACE2 and TMPRSS2 and is blocked by a clinically proven protease inhibitor. Cell 2020;181:271–280.e8. 32142651 10.1016/j.cell.2020.02.052 PMC7102627

[3] Weir M. The renin-angiotensin-aldosterone system: a specific target for hypertension management. Am J Hypertens 1999;12:205–213. 10.1016/s0895-7061(99)00103-x 10619573

[4] Danilczyk U, Penninger JM. Angiotensin-converting enzyme II in the heart and the kidney. Circ Res 2006;98:463–471. 16514079 10.1161/01.RES.0000205761.22353.5f

[5] Grasselli G, Zangrillo A, Zanella A, Antonelli M, Cabrini L, Castelli A, Cereda D, Coluccello A, Foti G, Fumagalli R, Iotti G, Latronico N, Lorini L, Merler S, Natalini G, Piatti A, Ranieri MV, Scandroglio AM, Storti E, Cecconi M, Pesenti A, for the COVID-19 Lombardy ICU Network. Baseline characteristics and outcomes of 1591 patients infected with SARS-CoV-2 admitted to ICUs of the Lombardy Region, Italy. JAMA 2020;323:1574–1581. 32250385 10.1001/jama.2020.5394 PMC7136855

[6] Guan W, Ni Z, Hu Y, Liang W, Ou C, He J, Liu L, Shan H, Lei C, Hui DSC, Du B, Li L, Zeng G, Yuen KY, Chen R, Tang C, Wang T, Chen P, Xiang J, Li S, Wang JL, Liang Z, Peng Y, Wei L, Liu Y, Hu YH, Peng P, Wang JM, Liu J, Chen Z, Li G, Zheng Z, Qiu S, Luo J, Ye C, Zhu S, Zhong N. Clinical characteristics of coronavirus disease 2019 in China. N Engl J Med 2020;382:1708–1720. 32109013 10.1056/NEJMoa2002032 PMC7092819

[7] Fang L, Karakiulakis G, Roth M. Are patients with hypertension and diabetes mellitus at increased risk for COVID-19 infection? Lancet Respir Med 2020;8:e21. 32171062 10.1016/S2213-2600(20)30116-8 PMC7118626

[8] Battistoni A, Volpe M. Might renin-angiotensin system blockers play a role in the COVID-19 pandemic? Eur Hear J Cardiovasc Pharmacother 2020;6:248–251. 10.1093/ehjcvp/pvaa030 PMC7184353 32286607

[9] Burchill LJ, Velkoska E, Dean RG, Griggs K, Patel SK, Burrell LM. Combination renin-angiotensin system blockade and angiotensin-converting enzyme 2 in experimental myocardial infarction: implications for future therapeutic directions. Clin Sci 2012;123:649–658. 10.1042/CS20120162 22715807

[10] Burrell LM, Burchill L, Dean RG, Griggs K, Patel SK, Velkoska E. Chronic kidney disease: cardiac and renal angiotensin-converting enzyme (ACE) 2 expression in rats after subtotal nephrectomy and the effect of ACE inhibition. Exp Physiol 2012;97:477–485. 22198016 10.1113/expphysiol.2011.063156

[11] Ramchand J, Patel SK, Kearney LG, Matalanis G, Farouque O, Srivastava PM, Burrell LM. Plasma ACE2 activity predicts mortality in aortic stenosis and is associated with severe myocardial fibrosis. JACC Cardiovasc Imaging 2020;13:655–664. 31607667 10.1016/j.jcmg.2019.09.005

[12] Ramchand J, Patel SK, Srivastava PM, Farouque O, Burrell LM. Elevated plasma angiotensin converting enzyme 2 activity is an independent predictor of major adverse cardiac events in patients with obstructive coronary artery disease. PLoS One 2018;13:e0198144. 29897923 10.1371/journal.pone.0198144 PMC5999069

[13] Monteil V, Kwon H, Prado P, Hagelkrüys A, Wimmer RA, Stahl M, Leopoldi A, Garreta E, Hurtado del Pozo C, Prosper F, Romero JP, Wirnsberger G, Zhang H, Slutsky AS, Conder R, Montserrat N, Mirazimi A, Penninger JM. Inhibition of SARS-CoV-2 infections in engineered human tissues using clinical-grade soluble human ACE2. Cell 2020;181:905–913.e7. 32333836 10.1016/j.cell.2020.04.004 PMC7181998

[14] Perico L, Benigni A, Remuzzi G. Should COVID-19 concern nephrologists? why and to what extent? The emerging impasse of angiotensin blockade. Nephron 2020;144:213–221. 32203970 10.1159/000507305 PMC7179544

[15] Murray E, Tomaszewski M, Guzik TJ. Binding of SARS-CoV-2 and angiotensin-converting enzyme 2: clinical implications. Cardiovasc Res 2020;116:e87–e89. 32301968 10.1093/cvr/cvaa096 PMC7184453

[16] Pan X. W, Xu D, Zhang H, Zhou W, Wang L. h, Cui X. G. Identification of a potential mechanism of acute kidney injury during the COVID-19 outbreak: a study based on single-cell transcriptome analysis. Intensive Care Med 2020;46:1114–1116. 32236644 10.1007/s00134-020-06026-1 PMC7106051

[17] Puelles VG, Lütgehetmann M, Lindenmeyer MT, Sperhake JP, Wong MN, Allweiss L, Chilla S, Heinemann A, Wanner N, Liu S, Braun F, Lu S, Pfefferle S, Schröder AS, Edler C, Gross O, Glatzel M, Wichmann D, Wiech T, Kluge S, Pueschel K, Aepfelbacher M, Huber TB. Multiorgan and renal tropism of SARS-CoV-2. N Engl J Med 2020;383:590–592. 32402155 10.1056/NEJMc2011400 PMC7240771

[18] Marques FZ, Romaine SPR, Denniff M, Eales J, Dormer J, Garrelds IM, Wojnar L, Musialik K, Duda-Raszewska B, Kiszka B, Duda M, Morris BJ, Samani NJ, Danser AHJ, Bogdanski P, Zukowska-Szczechowska E, Charchar FJ, Tomaszewski M. Signatures of miR-181a on the renal transcriptome and blood pressure. Mol Med 2015;21:739–748. 26322847 10.2119/molmed.2015.00096 PMC4818264

[19] Tomaszewski M, Eales J, Denniff M, Myers S, Chew GS, Nelson CP, Christofidou P, Desai A, Büsst C, Wojnar L, Musialik K, Jozwiak J, Debiec R, Dominiczak AF, Navis G, Gilst WV, Harst PVD, Samani NJ, Harrap S, Bogdanski P, Zukowska-Szczechowska E, Charchar FJ. Renal mechanisms of association between fibroblast growth factor 1 and blood pressure. J Am Soc Nephrol 2015;26:3151–3160. 25918036 10.1681/ASN.2014121211 PMC4657842

[20] Xu X, Eales JM, Akbarov A, Guo H, Becker L, Talavera D, Ashraf F, Nawaz J, Pramanik S, Bowes J, Jiang X, Dormer J, Denniff M, Antczak A, Szulinska M, Wise I, Prestes PR, Glyda M, Bogdanski P, Zukowska-Szczechowska E, Berzuini C, Woolf AS, Samani NJ, Charchar FJ, Tomaszewski M. Molecular insights into genome-wide association studies of chronic kidney disease-defining traits. Nat Commun 2018;9:4800. 30467309 10.1038/s41467-018-07260-4 PMC6250666

[21] Rowland J, Akbarov A, Eales J, Xu X, Dormer JP, Guo H, Denniff M, Jiang X, Ranjzad P, Nazgiewicz A, Prestes PR, Antczak A, Szulinska M, Wise IA, Zukowska-Szczechowska E, Bogdanski P, Woolf AS, Samani NJ, Charchar FJ, Tomaszewski M. Uncovering genetic mechanisms of kidney aging through transcriptomics, genomics, and epigenomics. Kidney Int 2019;95:624–635. 30784661 10.1016/j.kint.2018.10.029 PMC6390171

[22] Morris AP, Le TH, Wu H, Akbarov A, Most P. V D, Hemani G, Smith GD, Mahajan A, Gaulton KJ, Nadkarni GN, Valladares-Salgado A, Wacher-Rodarte N, Mychaleckyj JC, Dueker ND, Guo X, Hai Y, Haessler J, Kamatani Y, Stilp AM, Zhu G, Cook JP, Ärnlöv J, Blanton SH, Borst M. D, Bottinger EP, Buchanan TA, Cechova S, Charchar FJ, Chu PL, Damman J, Eales J, Gharavi AG, Giedraitis V, Heath AC, Ipp E, Kiryluk K, Kramer HJ, Kubo M, Larsson A, Lindgren CM, Lu Y, Madden PAF, Montgomery GW, Papanicolaou GJ, Raffel LJ, Sacco RL, Sanchez E, Stark H, Sundstrom J, Taylor KD, Xiang AH, Zivkovic A, Lind L, Ingelsson E, Martin NG, Whitfield JB, Cai J, Laurie CC, Okada Y, Matsuda K, Kooperberg C, Chen YDI, Rundek T, Rich SS, Loos RJF, Parra EJ, Cruz M, Rotter JI, Snieder H, Tomaszewski M, Humphreys BD, Franceschini N. Trans-ethnic kidney function association study reveals putative causal genes and effects on kidney-specific disease aetiologies. Nat Commun 2019;10:29. 30604766 10.1038/s41467-018-07867-7 PMC6318312

[23] Leek JT, Johnson WE, Parker HS, Jaffe AE, Storey JD. The SVA package for removing batch effects and other unwanted variation in high-throughput experiments. Bioinformatics 2012;28:882–883. 22257669 10.1093/bioinformatics/bts034 PMC3307112

[24] Beckerman P, Qiu C, Park J, Ledo N, Ko YA, Park ASD, Han SY, Choi P, Palmer M, Susztak K. Human kidney tubule-specific gene expression based dissection of chronic kidney disease traits. EBioMedicine 2017;24:267–276. 28970079 10.1016/j.ebiom.2017.09.014 PMC5652292

[25] Gamazon ER, Segrè AV, Bunt MVD, Wen X, Xi HS, Hormozdiari F, Ongen H, Konkashbaev A, Derks EM, Aguet F, Quan J, Nicolae DL, Eskin E, Kellis M, Getz G, McCarthy MI, Dermitzakis ET, Cox NJ, Ardlie KG, GTEx Consortium. Using an atlas of gene regulation across 44 human tissues to inform complex disease- and trait-associated variation. Nat Genet 2018;50:956–967. 29955180 10.1038/s41588-018-0154-4 PMC6248311

[26] Young MD, Mitchell TJ, Vieira Braga FA, Tran MGB, Stewart BJ, Ferdinand JR, Collord G, Botting RA, Popescu DM, Loudon KW, Vento-Tormo R, Stephenson E, Cagan A, Farndon SJ, Velasco-Herrera MDC, Guzzo C, Richoz N, Mamanova L, Aho T, Armitage JN, Riddick ACP, Mushtaq I, Farrell S, Rampling D, Nicholson J, Filby A, Burge J, Lisgo S, Maxwell PH, Lindsay S, Warren AY, Stewart GD, Sebire N, Coleman N, Haniffa M, Teichmann SA, Clatworthy M, Behjati S. Single-cell transcriptomes from human kidneys reveal the cellular identity of renal tumors. Science 2018;361:594–599. 30093597 10.1126/science.aat1699 PMC6104812

[27] Wakahara S, Konoshita T, Mizuno S, Motomura M, Aoyama C, Makino Y, Kato N, Koni I, Miyamori I. Synergistic expression of angiotensin-converting enzyme (ACE) and ACE2 in human renal tissue and confounding effects of hypertension on the ACE to ACE2 ratio. Endocrinology 2007;148:2453–2457. 17303661 10.1210/en.2006-1287

[28] Tukiainen T, Villani AC, Yen A, Rivas MA, Marshall JL, Satija R, Aguirre M, Gauthier L, Fleharty M, Kirby A, Cummings BB, Castel SE, Karczewski KJ, Aguet F, Byrnes A, Regev A, Ardlie KG, Hacohen N, MacArthur DG, GTEx Consortium; ; Laboratory, Data Analysis & Coordinating Center (LDACC)—Analysis Working Group; Statistical Methods groups—Analysis Working Group; Enhancing GTEx (eGTEx) groups; NIH Common Fund; NIH/NCI; NIH/NHGRI; NIH/NIMH; NIH/NIDA; Biospecimen Collection Source Site—NDRI; Biospecimen Collection Source Site—RPCI; Biospecimen Core Resource—VARI; Brain Bank Repository—University of Miami Brain Endowment Bank; Leidos Biomedical—Project Management; ELSI Study; Genome Browser Data Integration &Visualization—EBI; Genome Browser Data Integration &Visualization—UCSC Genomics Institute, University of California Santa Cruz; Tuuli Lappalainen. Landscape of X chromosome inactivation across human tissues. Nature 2017;550:244–248.29022598

[29] Varga Z, Flammer AJ, Steiger P, Haberecker M, Andermatt R, Zinkernagel AS, Mehra MR, Schuepbach RA, Ruschitzka F, Moch H. Endothelial cell infection and endotheliitis in COVID-19. Lancet 2020;395:1417–1418. 32325026 10.1016/S0140-6736(20)30937-5 PMC7172722

[30] Williams B, Zhang Y. Hypertension, renin–angiotensin–aldosterone system inhibition, and COVID-19. Lancet 2020;395:1671–1673. 32416786 10.1016/S0140-6736(20)31131-4 PMC7255199

[31] Farkash EA, Wilson AM, Jentzen JM. Ultrastructural evidence for direct renal infection with SARS-CoV-2. J Am Soc Nephrol 2020;31:1683–1687. 32371536 10.1681/ASN.2020040432 PMC7460898

[32] Su H, Yang M, Wan C, Yi LX, Tang F, Zhu HY, Yi F, Yang HC, Fogo AB, Nie X, Zhang C. Renal histopathological analysis of 26 postmortem findings of patients with COVID-19 in China. Kidney Int 2020;98:219–227. 32327202 10.1016/j.kint.2020.04.003 PMC7194105

[33] Batlle D, Soler MJ, Sparks MA, Hiremath S, South AM, Welling PA, Swaminathan S, on behalf of the COVID-19 and ACE2 in Cardiovascular, Lung, and Kidney Working Group. Acute kidney injury in COVID-19: emerging evidence of a distinct pathophysiology. J Am Soc Nephrol 2020;31:1380–1383. 32366514 10.1681/ASN.2020040419 PMC7350999

[34] Allison SJ. SARS-CoV-2 infection of kidney organoids prevented with soluble human ACE2. Nat Rev Nephrol 2020;16:316. 10.1038/s41581-020-0291-8 PMC7187150 32332922

[35] Clarke NE, Turner AJ. Angiotensin-converting enzyme 2: the first decade. Int J Hypertens 2012;2012:1–12. 10.1155/2012/307315 PMC3216391 22121476

[36] Abassi Z, Assady S, Khoury EE, Heyman SN. Letter to the editor: angiotensin-converting enzyme 2: an ally or a Trojan horse? Implications to SARS-CoV-2-related cardiovascular complications. Am J Physiol Heart Circ Physiol 2020;318:H1080–H1083. 32223552 10.1152/ajpheart.00215.2020 PMC7191629

[37] Kuba K, Imai Y, Ohto-Nakanishi T, Penninger JM. Trilogy of ACE2: a peptidase in the renin-angiotensin system, a SARS receptor, and a partner for amino acid transporters. Pharmacol Ther 2010;128:119–128. 20599443 10.1016/j.pharmthera.2010.06.003 PMC7112678

[38] Kowalczuk S, Bröer A, Tietze N, Vanslambrouck JM, Rasko JEJ, Bröer S. A protein complex in the brush‐border membrane explains a Hartnup disorder allele. FASEB J 2008;22:2880–2887. 18424768 10.1096/fj.08-107300

[39] Tikellis C, Thomas MC. Angiotensin-converting enzyme 2 (ACE2) is a key modulator of the renin angiotensin system in health and disease. Int J Pept 2012;2012:1–8. 10.1155/2012/256294 PMC3321295 22536270

[40] Archer SL, Sharp WW, Weir EK. Differentiating COVID-19 pneumonia from acute respiratory distress syndrome and high altitude pulmonary edema: therapeutic implications. Circulation 2020;142:101–104. 32369390 10.1161/CIRCULATIONAHA.120.047915 PMC7363563

[41] Nielsen R, Christensen EI, Birn H. Megalin and cubilin in proximal tubule protein reabsorption: from experimental models to human disease. Kidney Int 2016;89:58–67. 26759048 10.1016/j.kint.2015.11.007

[42] Tian E, Wang S, Zhang L, Zhang Y, Malicdan MC, Mao Y, Christoffersen C, Tabak LA, Schjoldager KT, Hagen KT. Galnt11 regulates kidney function by glycosylating the endocytosis receptor megalin to modulate ligand binding. Proc Natl Acad Sci U S A 2019;116:25196–25202. 31740596 10.1073/pnas.1909573116 PMC6911204

[43] Reich HN, Oudit GY, Penninger JM, Scholey JW, Herzenberg AM. Decreased glomerular and tubular expression of ACE2 in patients with type 2 diabetes and kidney disease. Kidney Int 2008;74:1610–1616. 19034303 10.1038/ki.2008.497

[44] Patel SK, Velkoska E, Freeman M, Wai B, Lancefield TF, Burrell LM. From gene to protein-experimental and clinical studies of ACE2 in blood pressure control and arterial hypertension. Front Physiol 2014;5:227. 25009501 10.3389/fphys.2014.00227 PMC4067757

[45] Bunyavanich S, Do A, Vicencio A. Nasal gene expression of angiotensin-converting enzyme 2 in children and adults. JAMA 2020;323:2427–2429. 32432657 10.1001/jama.2020.8707 PMC7240631

[46] Vaduganathan M, Vardeny O, Michel T, McMurray JJV, Pfeffer MA, Solomon SD. Renin-angiotensin-aldosterone system inhibitors in patients with COVID-19. N Engl J Med 2020;382:1653–1659. 32227760 10.1056/NEJMsr2005760 PMC7121452

[47] Ferrario CM, Jessup J, Chappell MC, Averill DB, Brosnihan KB, Tallant EA, Diz DI, Gallagher PE. Effect of angiotensin-converting enzyme inhibition and angiotensin II receptor blockers on cardiac angiotensin-converting enzyme 2. Circulation 2005;111:2605–2610. 15897343 10.1161/CIRCULATIONAHA.104.510461

[48] South AM, Tomlinson L, Edmonston D, Hiremath S, Sparks MA. Controversies of renin-angiotensin system inhibition during the COVID-19 pandemic. Nat Rev Nephrol 2020;16:305–307. 32246101 10.1038/s41581-020-0279-4 PMC7118703

[49] Guzik TJ, Mohiddin SA, Dimarco A, Patel V, Savvatis K, Marelli-Berg FM, Madhur MS, Tomaszewski M, Maffia P, D’Acquisto F, Nicklin SA, Marian AJ, Nosalski R, Murray EC, Guzik B, Berry C, Touyz RM, Kreutz R, Wang DW, Bhella D, Sagliocco O, Crea F, Thomson EC, McInnes IB. COVID-19 and the cardiovascular system: implications for risk assessment, diagnosis, and treatment options. Cardiovasc Res 2020;116:1666–1687. 32352535 10.1093/cvr/cvaa106 PMC7197627

[50] Deshotels MR, Xia H, Sriramula S, Lazartigues E, Filipeanu CM. Angiotensin II mediates angiotensin converting enzyme type 2 internalization and degradation through an Angiotensin II type I receptor-dependent mechanism. Hypertension 2014;64:1368–1375. 25225202 10.1161/HYPERTENSIONAHA.114.03743 PMC4231883

[51] Ishiyama Y, Gallagher PE, Averill DB, Tallant EA, Brosnihan KB, Ferrario CM. Upregulation of angiotensin-converting enzyme 2 after myocardial infarction by blockade of angiotensin II receptors. Hypertension 2004;43:970–976. 15007027 10.1161/01.HYP.0000124667.34652.1a

[52] Klimas J, Olvedy M, Ochodnicka-Mackovicova K, Kruzliak P, Cacanyiova S, Kristek F, Krenek P, Ochodnicky P. Perinatally administered losartan augments renal ACE2 expression but not cardiac or renal Mas receptor in spontaneously hypertensive rats. J Cell Mol Med 2015;19:1965–1974. 25766467 10.1111/jcmm.12573 PMC4549047

[53] Sama IE, Ravera A, Santema BT, Goor H. V, Maaten JT, Cleland JGF, Rienstra M, Friedrich AW, Samani NJ, Ng LL, Dickstein K, Lang CC, Filippatos G, Anker SD, Ponikowski P, Metra M, Veldhuisen D. V, Voors AA. Circulating plasma concentrations of angiotensin-converting enzyme 2 in men and women with heart failure and effects of renin-angiotensin-aldosterone inhibitors. Eur Heart J 2020;41:1810–1817. 32388565 10.1093/eurheartj/ehaa373 PMC7239195

[54] Walters TE, Kalman JM, Patel SK, Mearns M, Velkoska E, Burrell LM. Angiotensin converting enzyme 2 activity and human atrial fibrillation: increased plasma angiotensin converting enzyme 2 activity is associated with atrial fibrillation and more advanced left atrial structural remodelling. Europace 2017;19:1280–1287. 27738071 10.1093/europace/euw246

[55] Roberts MA, Velkoska E, Ierino FL, Burrell LM. Angiotensin-converting enzyme 2 activity in patients with chronic kidney disease. Nephrol Dial Transplant 2013;28:2287–2294. 23535224 10.1093/ndt/gft038 PMC7537611

[56] Hamming I, Timens W, Bulthuis MLC, Lely AT, Navis GJ, Goor HV. Tissue distribution of ACE2 protein, the functional receptor for SARS coronavirus. A first step in understanding SARS pathogenesis. J Pathol 2004;203:631–637. 15141377 10.1002/path.1570 PMC7167720

[57] Batlle D, Wysocki J, Soler MJ, Ranganath K. Angiotensin-converting enzyme 2: enhancing the degradation of angiotensin II as a potential therapy for diabetic nephropathy. Kidney Int 2012;81:520–528. 22113528 10.1038/ki.2011.381

[58] Mackey K, King VJ, Gurley S, Kiefer M, Liederbauer E, Vela K, Sonnen P, Kansagara D. Risks and impact of angiotensin-converting enzyme inhibitors or angiotensin-receptor blockers on SARS-CoV-2 infection in adults. Ann Intern Med 2020;173:195–203. 32422062 10.7326/M20-1515 PMC7249560

[59] Simone GD. Position statement of the ESC council on hypertension on ACE-inhibitors and angiotensin receptor blockers. Eur Soc Cardiol 2020. https://www.escardio.org/Councils/Council-on-Hypertension-(CHT)/News/position-statement-of-the-esc-council-on-hypertension-on-ace-inhibitors-and-ang (19 September 2020).

