# Supplementary material for: Hypertension and renin-angiotensin system blockers are not associated with expression of angiotensin-converting enzyme 2 (ACE2) in the kidney
Source: Eur Heart J. 2020 Oct 27;41(48):4580–8. doi: 10.1093/eurheartj/ehaa794 (PMC7665509; doi:10.1093/eurheartj/ehaa794)
Supplement: ehaa794_Supplementary_Data [file ehaa794_supplementary_data.docx]

**Hypertension and renin-angiotensin system blockers are not associated with expression of Angiotensin Converting Enzyme 2 (ACE2) in the kidney**

**Supplementary materials**

Xiao Jiang*^1^, James Eales*^1^, David Scannali^1^, Alicja Nazgiewicz^1^, Priscilla Prestes^2^, Michelle Maier^2^, Matthew Denniff^3^, Xiaoguang Xu^1^, Sushant Saluja^1^, Eddie Cano-Gamez^4^, Wojciech Wystrychowski^5^, Monika Szulinska^6^, Andrzej Antczak^7^, Sean Byars^8,9^, Damian Skrypnik^6^, Maciej Glyda^10^,

Robert Król^5^, Joanna Zywiec^11^, Ewa Zukowska-Szczechowska^12^, Louise M. Burrell^13^,

Adrian S. Woolf^14,15^, Adam Greenstein^1,16^, Pawel Bogdanski^6^, Bernard Keavney^1,16^,

Andrew P. Morris^17^, Anthony Heagerty^1,16^, Bryan Williams^18^, Stephen B. Harrap^19^, Gosia Trynka^4^, Nilesh J. Samani^3,20^, Tomasz J. Guzik^21,22^, Fadi J. Charchar^2,3,19^, Maciej Tomaszewski^1,16^

^1^Division of Cardiovascular Sciences, Faculty of Medicine, Biology and Health, University of Manchester, Manchester, UK

^2^School of Health and Life Sciences, Federation University Australia, Ballarat, Victoria, Australia

^3^Department of Cardiovascular Sciences, University of Leicester, Leicester, UK

^4^Department of Cellular Genetics, Wellcome Sanger Institute, Cambridge, UK

^5^Department of General, Vascular and Transplant Surgery, Medical University of Silesia, Katowice, Poland

^6^Department of Treatment of Obesity, Metabolic Disorders and Clinical Dietetics, Poznan University of Medical Sciences, Poznan, Poland.

^7^Department of Urology and Uro-oncology, Karol Marcinkowski University of Medical Sciences, Poznan, Poland

^8^Centre for Systems Genomics, School of BioSciences, The University of Melbourne, Parkville, Victoria, Australia

^9^Department of Pathology, The University of Melbourne, Parkville, Victoria, Australia

^10^Department of Transplantology and General Surgery Poznan, Collegium Medicum, Nicolaus Copernicus University, Bydgoszcz, Poland

^11^Department of Internal Medicine, Diabetology and Nephrology, Medical University of Silesia, Zabrze, Poland

^12^Department of Health Care, Silesian Medical College, Katowice, Poland

^13^Department of Medicine and Cardiology, University of Melbourne, Austin Health, Melbourne, Victoria, Australia

^14^Division of Cell Matrix Biology and Regenerative Medicine, Faculty of Biology Medicine and Health, University of Manchester, Manchester, UK

^15^Royal Manchester Children’s Hospital and Manchester Academic Health Science Centre, Manchester University NHS Foundation Trust, Manchester, UK

^16^Division of Medicine and Manchester Academic Health Science Centre, Manchester University NHS Foundation Trust Manchester, Manchester, UK

^17^Division of Musculoskeletal & Dermatological Sciences, Faculty of Medicine, Biology and Health, University of Manchester, Manchester, UK

^18^Institute of Cardiovascular Sciences, University College London, London, UK

^19^Department of Physiology, University of Melbourne, Melbourne, Victoria, Australia

^20^National Institute for Health Research, Leicester Biomedical Research Centre, Leicester, UK

^21^Institute of Cardiovascular and Medical Sciences, College of Medical, Veterinary and Life Sciences, University of Glasgow, Glasgow, UK

^22^Department of Internal and Agricultural Medicine, Jagiellonian University College of Medicine, Kraków, Poland

*These authors contributed equally to this work

Table of Contents

[1. Methods 4](#_Toc48333917)

[1.1. Human kidney tissue collections 4](#_Toc48333918)

[1.2. Phenotypes 4](#_Toc48333919)

[1.3. Genotyping and genetic principal components 5](#_Toc48333920)

[1.4. Kidney transcriptome profiling 6](#_Toc48333921)

[1.5. Kidney single-cell RNA-sequencing analysis 7](#_Toc48333922)

[1.6. Kidney ACE2 co-expression analysis 7](#_Toc48333923)

[1.7. Renal expression of ACE2 – association analyses 8](#_Toc48333924)

[1.8. Expression of ACE2 in other human tissues – Genotype-Tissue Expression project (GTEx) 9](#_Toc48333925)

[1.9. Kidney expression of ACE2 and eGFR – replication analysis in independent datasets 9](#_Toc48333926)

[1.10. Kidney expression of ACE2 in rats treated with perindopril and losartan 10](#_Toc48333927)

[1.11. Bioethics 10](#_Toc48333928)

[2. Supplementary Tables 12](#_Toc48333929)

[2.1. Table S1. Demographic and clinical characteristics. 12](#_Toc48333930)

[2.2. Table S2. Analysis of association between renal expression of ACE2 and clinical phenotypes in the discovery resource – summary of results. 13](#_Toc48333931)

[2.3. Table S3. Linear correlation between renal tubulointerstitial ACE2 expression and eGFR in *Nephroseq*. 14](#_Toc48333932)

[3. Supplementary Figure. 15](#_Toc48333933)

[4. References 17](#_Toc48333934)

# 1. Methods

## Human kidney tissue collections

We used 436 human kidney samples collected from patients recruited into five studies [TRANScriptome of renaL humAn TissuE (TRANSLATE) study^1-5^, its extension – TRANSLATE-T study^3^, moleculAr analysis of human kiDney-Manchester renal tIssue pRojEct (ADMIRE), molecular analysis of mechanisms Regulating gene Expression in Post-ischAemic Injury to Renal allograft (REPAIR) and Renal gEne expreSsion and PredispOsition to cardiovascular and kidNey Disease (RESPOND)] in our discovery resource. In TRANSLATE, ADMIRE and RESPOND studies, the samples were collected from patients with unilateral kidney cancer – the specimen was taken from unaffected by cancer part of the kidney immediately after elective nephrectomy^1-5^. TRANSLATE-T and REPAIR collected pre-implantation kidney biopsies^3^ from deceased kidney donors prior to the organ transplantation^3^. The secured tissue samples from all the studies were immersed immediately in RNAlater or snap-frozen for the purpose of further molecular analysis. We used an additional cohort of 98 individuals from the NIH-supported Tissue Cancer Genome Atlas (TCGA)^6,7^ as a replication resource for some of our kidney gene expression analyses. We only used samples collected as “companion normal tissue specimen” – those taken from healthy (unaffected by cancer) part of the kidney after the surgery^7^, in line with the tissue collection strategy used by TRANSLATE study. All patients were of white-European ethnicity.

## Phenotypes

For the purpose of this project we extracted the relevant demographic and clinical information (age, sex, body weight index, height, blood pressure (BP), hypertension, diabetes, renal function) from the discovery projects’ databases. We calculated body mass index by dividing weight (in kg) by height squared (in m^2^). In TRANSLATE and RESPOND studies, hypertension was defined as BP values ≥140/90 mmHg (measured on at least two separate occasions) and/or being on pharmacological antihypertensive treatment, as reported elsewhere^2^. Diabetes was defined as either self-reported history of diabetes and/or being on hypoglycaemic medications^4^. In ADMIRE, TRANSLATE-T and REPAIR studies, information on history of hypertension and diabetes was recorded by the respective recruitment teams based on the available hospital documentation. All hypertensive patients had information on whether they were managed using BP lowering medications. A total of 72.4% of hypertensive patients on pharmacological antihypertensive treatment had further detailed information on the prescribed antihypertensive medications. In these patients we allocated each of the prescribed antihypertensive medication into one of the following antihypertensive classes: angiotensin converting enzyme inhibitors (ACE-I), angiotensin II type 1 receptor (AT1) antagonists (ARB), beta-blockers (BB), calcium channel antagonists (CCA), diuretics (DRT) or others. Serum levels of creatinine were measured by modified kinetic Jaffe method (calibrated to an IDMS reference measurement procedure) in 310 individuals. In calculating estimated glomerular filtration rate (eGFR) we used CKD-EPI equation^8^, as reported before^4^. Only basic demographic information (age, sex) were available for patients recruited in the kidney replication resource (TCGA)^4^.

## Genotyping and genetic principal components

In the discovery resource, kidney DNA was extracted using Qiagen DNeasyBlood and Tissue Kit, and then hybridised to the Infinium® HumanCoreExome-24 beadchip array, as reported before^3,5^. In TCGA, DNA was extracted from blood samples using QiAAmp Blood Midi Kit (CGARN, 2016) and hybridised with probes on the Affymetrix SNP 6.0 array^7^. We applied the same quality control filters to genotyped variants and individuals in all data sets. In brief, we excluded autosomal genotyped variants based on the following criteria: genotyping rate <95%, Hardy-Weinberg equilibrium (HWE) P<1x10^−3^, minor allele frequency (MAF)<5%, position duplicated variants^3^. Prior to further analyses we removed individuals with: genotyping rate <95%, heterozygosity rate outside ±3 standard deviations from the mean, cryptic relatedness to other individuals, inconsistent sex information and non-white-European genetic ancestry^3^. Genotype principal components (PCs) were calculated using PLINK v1.90b6.2^9^. In the calculation we firstly removed ambiguous genetic variants (C/G or A/T) with MAF>0.4. Genetic variants in 24 high linkage disequilibrium (LD) regions were removed thereafter. We then used “--indep-pairwise” in PLINK to further prune variants in LD setting window size of 1000bp, step size of 50 variants and r^2^ cut-off threshold as 0.05. We calculated the genotype PCs for each of the discovery and replication resource, using “--pca” in PLINK.

## Kidney transcriptome profiling

We used RNeasy kits (Qiagen) to extract RNA from kidney tissue in 75% of samples in our discovery resource. The remaining samples were subjected to an RNA extraction using miRNeasy Mini Kit (Qiagen). In the replication resource, kidney RNA was extracted from snap-frozen samples using a modification of the DNA/RNA AllPrep Kit (Qiagen), as reported before (https://brd.nci.nih.gov/brd/sop/show/1450).

Sequencing libraries were generated from 1µg of extracted RNA using Illumina TruSeq poly-A protocol. Libraries were then sequenced using either 100 bp reads (on an Illumina HiSeq 2000) or 75 bp paired-end reads (on an Illumina NextSeq or HiSeq 4000) producing an average of 32 million paired reads and 5.5 Gb per sample. The base calling and sequence quality were evaluated using FastQC^10^ on all sequenced libraries. We used Kallisto to quantify the expression of genes at a transcript level [in transcripts per million (TPM)]^11^ and then summed them to generate gene-level expression values. For the purpose of our downstream computational analyses we selected genes with the expression >TPM of 0.1 and the read count ≥6 (in at least 20% of kidney samples, within each study). All sequenced samples were examined using several quality control filters including: number of total reads (>10million reads), D-statistic test (a normalised measure of within tissue sample inter-correlation, D≤5), sex compatibility check (consistency between the reported sex and expression of sex-specific genes, based on XIST and expression of RPS4Y1, KDM5D, DDX3Y, EIF1AY and USP9Y), verification of sample code based on comparing DNA base calls obtained from RNA-sequencing using GATK and DNA genotype calls and visual inspection of principal component plots of processed TPM data.

After applying these quality control filters, 21,203 renal genes (common for all studies in the discovery and replication dataset) were retained for the downstream analyses. Prior to the downstream analyses, gene expression data that passed the quality control underwent normalisation. The expression values underwent first log-transformation [natural logarithm of TPM (plus an offset of 1)] followed by quantile normalisation (http://bioconductor.org/packages/release/bioc/html/aroma.light.html). The quantile-normalised data were then standardised using rank-based inverse normal transformation^3^.

## Kidney single-cell RNA-sequencing analysis

Single-cell RNA-sequencing data from the human adult kidney was obtained from Young et al.^12^ and re-analysed. Briefly, we used data from 41,778 normal kidney cells, combined this with literature-curated markers to annotate cell types and used Seurat^13^ to determine cell type marker genes and a two-dimensional t-distributed stochastic neighbour embedding (t-SNE) of the expression data. We identified 23 distinct cell clusters. These were then grouped into 8 renal cell types, 5 immune cell types and 1 unidentified by their respective expression of canonical cell type markers. The 8 renal cell types were then grouped and visualised by their localisation to each nephron segment.

## Kidney ACE2 co-expression analysis

The ACE2 co-expression analysis employed multivariate regression to determine the association between each expressed kidney gene and ACE2; models were adjusted for age, sex, diabetes, hypertension, three genetic PCs and surrogate variables automatically inferred by the “sva” R package in line with Tukiainen et al.^14^. This analytical pipeline was applied to the discovery and replication data sets separately. The crude P-values were then adjusted by permutation-based resampling. The crude t-statistics were scaled by the mean and standard deviation of t-statistics from 1,000 permutations of the data, then the P-values were re-calculated and the FDR-based adjustment for multiple testing was applied. A gene was considered as co-expressed with ACE2 if the following criteria were met: (i) FDR <0.05 in the discovery data, (ii) FDR <0.05 in the replication data and (iii) consistent direction of association with ACE2 in both discovery and replication.

Gene set overrepresentation for REACTOME pathways and gene ontology cellular compartment annotation was performed by the PANTHER classification system^15^ employing Fisher’s exact test. Official gene symbols for all ACE2 co-expressed genes were uploaded as an input list to the PANTHER analysis and were compared against all human genes. The kidney disease and BP gene sets were built from a combination of GWAS catalog (https://www.ebi.ac.uk/gwas/) association data for phenotypes “Blood pressure” (EFO_0004325) and “Kidney disease” (EFO_0003086) and manually curated genetic associations from the GWAS literature. The resulting sets contain genes known for their association with BP regulation or kidney health/disease (with the directionality of the effect dependent i.e. on the allele dosages or the magnitude of expression, etc.). Their enrichment among ACE2-coexpressed genes was examined using Fisher’s exact test and all human genes as a comparator.

## Renal expression of ACE2 – association analyses

We used ordinary least squares linear regression models to examine associations between normalised renal expression of ACE2 [or sex-related “control” genes (XIST, RPS4Y1)] as dependent variables with each of the selected demographic/clinical variables – age, sex, body mass index (BMI), clinic systolic and diastolic BP, hypertension, diabetes and eGFR as independent parameters; separately in the discovery and the replication resource (where available). All the regression models were adjusted for (i) first three genotype PCs – to adjust for the population structure, (ii) a set of demographic (age, sex) and clinical (i.e. BMI, diabetes; where appropriate) parameters – to minimise their potentially confounding effects on the dependent variable and (iii) surrogate variables (SVs) – to remove the unwanted effects such as these generated by variation in recruitment centres, DNA and RNA processing protocols, and times of sequencing^16^. The SVs also remove the variation from unmeasured cofounders, including but not limited to technical variables (sources of tissues, effects of differences in cell types^17^) as well as latent biological variables^16^ (i.e. environmental, genetic or clinical). The SVs were calculated for each model separately. The optimised number of surrogate variables for each model was determined by the “sva” package^16^, in line with previous studies^14,18^. The results from the analysis of the association between renal expression of ACE2 and sex in the discovery and replication resources were combined using an inverse-variance fixed-effect meta-analysis^19^.

The analysis of the association between renal expression of ACE2 and each of the antihypertensive class was adjusted for age, sex, BMI, diabetes, first three genotype PCs and SVs, and further corrected for the inter-correlation between the drug classes (using “gee” R package). The absolute normalised and adjusted differences in renal ACE2 between those who were taking a specific class of antihypertensive medications versus those who were not on any medication were converted into fold differences using the exponential values of the corresponding estimated coefficients. The adjustment for multiple testing was calculated using the “qvalue” package^20,21^ in R.

*A priori* calculations showed good [67-99.9%] power of our study to detect a one SD difference in expression of a kidney gene [whose average abundance and its dispersion was consistent with that of renal ACE2 in an independent collection of human kidneys^6,7^] between two groups and across a wide range of antihypertensive prescription frequency (from 5% to 50%) with a Bonferroni-adjusted level of significance (to account for the number of classes of antihypertensive medications examined and to maintain experiment-wise type I error of 5*%*). All computations were conducted by the R package “pwr” (using the two sample t-test power option and the two-sided alternative hypothesis).

## Expression of ACE2 in other human tissues – Genotype-Tissue Expression project (GTEx)

We also explored the gene expression information from Genotype-Tissue Expression project (GTEx) – an NIH-sponsored publicly available resource^22^. We extracted information on normalised ACE2 expression from the transcriptome of the lung as well as the following human tissues of relevance to hypertension/BP regulation: adrenal gland, visceral adipose tissue, subcutaneous adipose tissue, left ventricle, atrial appendage, tibial artery, coronary artery and aorta^22^. The number of post-mortem donors who contributed transcriptomic information for these tissues was 515, 233, 469, 581, 386, 372, 584, 213 and 387 (respectively). Analysis of association between ACE2 expression and age as well as sex were completed separately in each tissue using a multivariate regression model and the “Limma” R package^23^. All models were adjusted for age, sex (as appropriate), BMI, sample ischaemic time, death classification on the Hardy scale^14^, 3 genetic PCs and a tissue-specific number of SVs inferred by the “sva” R package. The nominal P-values were further adjusted by FDR.

## Kidney expression of ACE2 and eGFR – replication analysis in independent datasets

To replicate the association between kidney ACE2 expression and eGFR, we first examined renal gene expression data sets curated by *Nephroseq* (www.nephroseq.org). Included were gene expression studies on human renal tissue (tubulointerstitium) with a minimum of 10 informative individuals in the analyses. Five datasets from four studies [including European Renal cDNA Bank (ERCB), Ju et al. – discovery and validation^24^, Sampson et al.^25^, Woroniecka et al.^26^] were eligible; in ERCB transcriptome profiling was conducted using RNA-sequencing, in the other studies – by microarrays. For each of the studies we computed Pearson’s correlation coefficient for renal tubulointerstitial expression of ACE2 against eGFR. To determine the overall correlation between renal ACE2 expression and eGFR we then combined the information from all studies with the Olkin-Pratt fixed-effect meta-analytical approach (using the R package Metacor). Heterogeneity was examined using Cochran’s Q test. We then explored another microarray-derived dataset from 95 human renal tubule samples and extracted the results of adjusted (for age, sex and other clinical variables) association between ACE2 and eGFR^27^.

## Kidney expression of ACE2 in rats treated with perindopril and losartan

To examine ACE2 expression in response to losartan and perindopril, we first sourced 6-week old inbred male spontaneously hypertensive rats (SHR) from the Animal Resources Centre (Canning Vale, Western Australia). At 10 weeks old, SHR were implanted with minipumps (Alzet model 2004, Durect Corp Cupertino, California) for infusions of either the vehicle (normal saline), losartan (7.5 mg/kg/d) or perindopril (1 mg/kg/d). SHR received infusions for 4 weeks, after which treatment ceased at 14 weeks old. SHR were sacrificed at 14 weeks old (n=4-10) under general anaesthetic with isoflurane and ketamine. The kidneys were removed, decapsulated and dissected; renal cortices were subsequently submerged in RNAlater stabilisation solution (Thermo Fisher Scientific), frozen in liquid nitrogen and stored at -80°C. RNA was later extracted from the isolated cortices using the mirVana™ miRNA isolation kit (Thermo Fisher Scientific) and quantified using the NanoDrop 2000 (Thermo Fisher Scientific). To account for technical variation, gene expression of ACE2 was normalised against housekeeping gene (GAPDH) using quantitative real-time PCR. We then performed unpaired student t-tests on ΔCt values using GraphPad Prism 8 to assess variability between groups.

## Bioethics

The human studies adhered to the Declaration of Helsinki and were approved/ratified by the appropriate Bioethics Committee of the Medical University of Silesia (Katowice, Poland), Bioethics Committee of Karol Marcinkowski Medical University (Poznan, Poland), Ethics Committee of University of Leicester (Leicester, UK), the University of Manchester Research Ethics Committee (Manchester, UK) and the NHS Health Research Authority, National Research Ethics Service Committee – North West (Manchester, UK). Informed written consents were obtained from all individuals – for the deceased donors from TRANSLATE-T, the consent was obtained from the members of the family. All animal experiments were approved by the University of Melbourne Animal Ethics Committee and were conducted in accordance with the Australian Code of Practice for the Care and Use of Animals for Scientific Purposes.

# 2. Supplementary Tables

## 2.1. Table S1. Demographic and clinical characteristics.

| **Phenotype** | **Discovery** | | | | | | **Replication** |
| --- | --- | --- | --- | --- | --- | --- | --- |
|  | **All** | **ADMIRE** | **TRANSLATE** | **TRANSLATE-T** | **REPAIR** | **RESPOND** | **TCGA** |
| Number | 436 | 50 | 230 | 105 | 40 | 11 | 98 |
| Age (years) | 57.3 (14.1) | 66.0 (10.1) | 61.7 (10.8) | 47.0 (14.8) | 47.1 (13.8) | 62.6 (10.5) | 61.2 (13.2) |
| Female | 162 (37.2%) | 16 (32.0%) | 89 (38.7%) | 41 (39.0%) | 13 (32.5%) | 3 (27.3%) | 29 (29.6%) |
| Body mass index (kg/m^2^) | 27.5 (4.9) | 28.2 (5.5) | 28.2 (5.0) | 26.3 (4.9) | 26.4 (3.5) | 27.1 (2.0) | - |
| Hypertension | 269 (61.7%) | 28 (56.0%) | 155 (67.4%) | 63 (60.0%) | 13 (32.5%) | 10 (90.9%) | - |
| Diabetes | 57 (13.1%) | 8 (16.0%) | 37 (16.1%) | 8 (7.6%) | 1 (2.5%) | 3 (27.3%) | - |
| eGFR  (ml/min/1.73 m^2^) | 75.1 (21.1) | 67.0 (11.6) | 77.7 (19.2) | 71.4 (30.3) | - | - | - |

Data are counts and percentages or means and standard deviations. eGFR – estimated Glomerular Filtration Rate; ADMIRE – moleculAr analysis of human kiDney-Manchester renal tIssue pRojEct, TRANSLATE – TRANScriptome of renaL humAn TissuE study, TRANSLATE-T – TRANSLATE transplantation extension, REPAIR - molecular analysis of mechanisms Regulating gene Expression in Post-ischAemic Injury to Renal allograft stuudy, RESPOND – Renal gEne expreSsion and PredispOsition to cardiovascular and kidNey Disease study, TCGA – Tissue Cancer Genome Atlas.

## 2.2. Table S2. Analysis of association between renal expression of ACE2 and clinical phenotypes in the discovery resource – summary of results.

| **No** | **Phenotype** | **N** | **P-value** |
| --- | --- | --- | --- |
| 1. | Hypertension | 436 | 0.6008 |
| 2 | Hypertension* | 215 | 0.1212 |
| 3 | Diabetes | 436 | 0.8445 |
| 4 | Body mass index | 436 | 0.8843 |
| 5 | eGFR | 310 | 2.41x10^-5^ |

N – number of informative subjects, Hypertension* – sensitivity analysis restricted to those not on antihypertensive treatment, eGFR – estimated Glomerular Filtration Rate, P-value – level of statistical significance from the regression models

## 2.3. Table S3. Linear correlation between renal tubulointerstitial ACE2 expression and eGFR in *Nephroseq*.

| **Study** | **Number of patients** | **Number of controls** | **Total number of subjects** | **Number of available samples** | **r** | **P-value** | **Reference** |
| --- | --- | --- | --- | --- | --- | --- | --- |
| Ju et al. - discovery | 170 | 31 | 201 | 186 | 0.386 | 5.2x10^-8^ | Sci Transl Med. 2015;7:316. |
| European Renal cDNA Bank | 28 | 9 | 37 | 16 | 0.784 | 3.22x10^-4^ | RNA-Seq of 18 samples with focal segmental glomerulosclerosis, 10 – with diabetic nephropathy, and 9 healthy living donor samples |
| Sampson et al. | n/a | n/a | 50 | 49 | 0.367 | 0.009 | J Am Soc Nephrol. 2016;27:814-23. |
| Woroniecka et al. | 10 | 12 | 22 | 22 | 0.477 | 0.025 | Diabetes. 2011;60:2354-69. |
| Ju et al. - validation | n/a | n/a | 42 | 42 | 0.315 | 0.042 | Sci Transl Med. 2015;7(316). |
|  | | | | | **meta-r: 0.403** | **meta-P: 1.19x10^-17^** |  |

eGFR – estimated Glomerular Filtration Rate, n/a – data unavailable, r – Pearson’s correlation between renal tubulointerstitial ACE2 expression and eGFR, P-value – level of statistical significance, meta-r – meta-analysed correlation coefficient, meta-P – level of statistical significance for the meta-analysed correlation.

3. Supplementary Figure.

**A.** Differences in renal expression of the sex-specific control genes (XIST – X inactive specific transcript and RPS4Y1 – Ribosomal protein S4, Y-linked 1) between men and women in the discovery and the replication datasets. Blue – higher renal expression in males than females; Red – higher renal expression in females than males. **B.** Difference in renal expression of ACE2 between men and women in the discovery dataset. RBINT – the residual of normalised ACE2 expression, n – number of individuals. **C.** Difference in renal expression of ACE2 between men and women in the replication dataset. **D.** Differences in renal expression of ACE2 between men and women – a meta-analysis of both the discovery and replication dataset. n – number of individuals, FC – fold change, 95% CI – 95% confidence interval, P-value – level of statistical significance, F vs M – fold-difference (female-male) of renal ACE2 expression. **E.** Association between lung expression of ACE2 and age in the Genotype-Tissue Expression project (GTEx). **F.** Differences in renal expression of ACE2 between hypertension and normotension – sensitivity analysis restricted to patients not taking antihypertensive medications. **G.** Differences in renal expression of ACE2 between diabetic and non-diabetic individuals. **H.** Percentages of patients on different classes of antihypertensive drugs from the discovery resource – information based on 160 patients with available information on prescribed blood pressure lowering therapy. ACE-I – angiotensin converting enzyme inhibitors, ARB – angiotensin II type 1 receptor antagonists, BB – beta-blockers, CCA – calcium channel antagonists, DRT – diuretics, others – other antihypertensive drugs. **I.** Meta-analysis of linear correlation between renal tubulointerstitial ACE2 expression and eGFR in *Nephroseq*. X-axis – direction of correlation, ERCB – European Renal cDNA Bank, n – number of informative individuals, r – Pearson’s correlation, P-value – level of statistical significance.

# 4. References

1. Marques FZ, Romaine SPR, Denniff M, Eales J, Dormer J, Garrelds IM, Wojnar L, Musialik K, Duda-Raszewska B, Kiszka B, Duda M, Morris BJ, Samani NJ, Jan Danser AH, Bogdanski P, Zukowska-Szczechowska E, Charchar FJ, Tomaszewski M. Signatures of miR-181a on the renal transcriptome and blood pressure. *Mol Med* 2015;**21**:739–748.

2. Tomaszewski M, Eales J, Denniff M, Myers S, Chew GS, Nelson CP, Christofidou P, Desai A, Büsst C, Wojnar L, Musialik K, Jozwiak J, Debiec R, Dominiczak AF, Navis G, Gilst WH Van, Harst P Van Der, Samani NJ, Harrap S, Bogdanski P, Zukowska-Szczechowska E, Charchar FJ. Renal mechanisms of association between fibroblast growth factor 1 and blood pressure. *J Am Soc Nephrol* 2015;**26**:3151–3160.

3. Xu X, Eales JM, Akbarov A, Guo H, Becker L, Talavera D, Ashraf F, Nawaz J, Pramanik S, Bowes J, Jiang X, Dormer J, Denniff M, Antczak A, Szulinska M, Wise I, Prestes PR, Glyda M, Bogdanski P, Zukowska-Szczechowska E, Berzuini C, Woolf AS, Samani NJ, Charchar FJ, Tomaszewski M. Molecular insights into genome-wide association studies of chronic kidney disease-defining traits. *Nat Commun* 2018;**9**:4800.

4. Rowland J, Akbarov A, Eales J, Xu X, Dormer JP, Guo H, Denniff M, Jiang X, Ranjzad P, Nazgiewicz A, Prestes PR, Antczak A, Szulinska M, Wise IA, Zukowska-Szczechowska E, Bogdanski P, Woolf AS, Samani NJ, Charchar FJ, Tomaszewski M. Uncovering genetic mechanisms of kidney aging through transcriptomics, genomics, and epigenomics. *Kidney Int* 2019;**95**:624–635.

5. Morris AP, Le TH, Wu H, Akbarov A, Most PJ van der, Hemani G, Smith GD, Mahajan A, Gaulton KJ, Nadkarni GN, Valladares-Salgado A, Wacher-Rodarte N, Mychaleckyj JC, Dueker ND, Guo X, Hai Y, Haessler J, Kamatani Y, Stilp AM, Zhu G, Cook JP, Ärnlöv J, Blanton SH, Borst MH de, Bottinger EP, Buchanan TA, Cechova S, Charchar FJ, Chu PL, Damman J, Eales J, Gharavi AG, Giedraitis V, Heath AC, Ipp E, Kiryluk K, Kramer HJ, Kubo M, Larsson A, Lindgren CM, Lu Y, Madden PAF, Montgomery GW, Papanicolaou GJ, Raffel LJ, Sacco RL, Sanchez E, Stark H, Sundstrom J, Taylor KD, Xiang AH, Zivkovic A, Lind L, Ingelsson E, Martin NG, Whitfield JB, Cai J, Laurie CC, Okada Y, Matsuda K, Kooperberg C, Chen YDI, Rundek T, Rich SS, Loos RJF, Parra EJ, Cruz M, Rotter JI, Snieder H, Tomaszewski M, Humphreys BD, Franceschini N. Trans-ethnic kidney function association study reveals putative causal genes and effects on kidney-specific disease aetiologies. *Nat Commun* 2019;**10**:29.

6. Weinstein JN, Collisson EA, Mills GB, Shaw KRM, Ozenberger BA, Ellrott K, Sander C, Stuart JM, Chang K, Creighton CJ, Davis C, Donehower L, Drummond J, Wheeler D, Ally A, Balasundaram M, Birol I, Butterfield YSN, Chu A, Chuah E, Chun HJE, Dhalla N, Guin R, Hirst M, Hirst C, Holt RA, Jones SJM, Lee D, Li HI, Marra MA, Mayo M, Moore RA, Mungall AJ, Robertson AG, Schein JE, Sipahimalani P, Tam A, Thiessen N, Varhol RJ, Beroukhim R, Bhatt AS, Brooks AN, Cherniack AD, Freeman SS, Gabriel SB, Helman E, Jung J, Meyerson M, Ojesina AI, Pedamallu CS, Saksena G, Schumacher SE, Tabak B, Zack T, Lander ES, Bristow CA, Hadjipanayis A, Haseley P, Kucherlapati R, Lee S, Lee E, Luquette LJ, Mahadeshwar HS, Pantazi A, Parfenov M, Park PJ, Protopopov A, Ren X, Santoso N, Seidman J, Seth S, Song X, Tang J, Xi R, Xu AW, Yang L, Zeng D, Auman JT, Balu S, Buda E, Fan C, Hoadley KA, Jones CD, Meng S, Mieczkowski PA, Parker JS, Perou CM, Roach J, Shi Y, Silva GO, Tan D, Veluvolu U, Waring S, Wilkerson MD, Wu J, Zhao W, Bodenheimer T, Hayes DN, Hoyle AP, Jeffreys SR, Mose LE, Simons J V., Soloway MG, Baylin SB, Berman BP, Bootwalla MS, Danilova L, Herman JG, Hinoue T, Laird PW, Rhie SK, Shen H, Triche T, Weisenberger DJ, Carter SL, Cibulskis K, Chin L, Zhang J, Sougnez C, Wang M, Getz G, Dinh H, Doddapaneni HV, Gibbs R, Gunaratne P, Han Y, Kalra D, Kovar C, Lewis L, Morgan M, Morton D, Muzny D, Reid J, Xi L, Cho J, Dicara D, Frazer S, Gehlenborg N, Heiman DI, Kim J, Lawrence MS, Lin P, Liu Y, Noble MS, Stojanov P, Voet D, Zhang H, Zou L, Stewart C, Bernard B, Bressler R, Eakin A, Iype L, Knijnenburg T, Kramer R, Kreisberg R, Leinonen K, Lin J, Liu Y, Miller M, Reynolds SM, Rovira H, Shmulevich I, Thorsson V, Yang D, Zhang W, Amin S, Wu CJ, Wu CC, Akbani R, Aldape K, Baggerly KA, Broom B, Casasent TD, Cleland J, Dodda D, Edgerton M, Han L, Herbrich SM, Ju Z, Kim H, Lerner S, Li J, Liang H, Liu W, Lorenzi PL, Lu Y, Melott J, Nguyen L, Su X, Verhaak R, Wang W, Wong A, Yang Y, Yao J, Yao R, Yoshihara K, Yuan Y, Yung AK, Zhang N, Zheng S, Ryan M, Kane DW, Aksoy BA, Ciriello G, Dresdner G, Gao J, Gross B, Jacobsen A, Kahles A, Ladanyi M, Lee W, Lehmann K Van, Miller ML, Ramirez R, Rätsch G, Reva B, Schultz N, Senbabaoglu Y, Shen R, Sinha R, Sumer SO, Sun Y, Taylor BS, Weinhold N, Fei S, Spellman P, Benz C, Carlin D, Cline M, Craft B, Goldman M, Haussler D, Ma S, Ng S, Paull E, Radenbaugh A, Salama S, Sokolov A, Swatloski T, Uzunangelov V, Waltman P, Yau C, Zhu J, Hamilton SR, Abbott S, Abbott R, Dees ND, Delehaunty K, Ding L, Dooling DJ, Eldred JM, Fronick CC, Fulton R, Fulton LL, Kalicki-Veizer J, Kanchi KL, Kandoth C, Koboldt DC, Larson DE, Ley TJ, Lin L, Lu C, Magrini VJ, Mardis ER, McLellan MD, McMichael JF, Miller CA, O’Laughlin M, Pohl C, Schmidt H, Smith SM, Walker J, Wallis JW, Wendl MC, Wilson RK, Wylie T, Zhang Q, Burton R, Jensen MA, Kahn A, Pihl T, Pot D, Wan Y, Levine DA, Black AD, Bowen J, Frick J, Gastier-Foster JM, Harper HA, Helsel C, Leraas KM, Lichtenberg TM, McAllister C, Ramirez NC, Sharpe S, Wise L, Zmuda E, Chanock SJ, Davidsen T, Demchok JA, Eley G, Felau I, Sheth M, Sofia H, Staudt L, Tarnuzzer R, Wang Z, Yang L, Zhang J, Omberg L, Margolin A, Raphael BJ, Vandin F, Wu HT, Leiserson MDM, Benz SC, Vaske CJ, Noushmehr H, Wolf D, Veer LVT, Anastassiou D, Yang THO, Lopez-Bigas N, Gonzalez-Perez A, Tamborero D, Xia Z, Li W, Cho DY, Przytycka T, Hamilton M, McGuire S, Nelander S, Johansson P, Jörnsten R, Kling T. The cancer genome atlas pan-cancer analysis project. *Nat Genet* 2013;**45**:1113–1120.

7. Creighton CJ, Morgan M, Gunaratne PH, Wheeler DA, Gibbs RA, Robertson G, Chu A, Beroukhim R, Cibulskis K, Signoretti S, Vandin F, Wu HT, Raphael BJ, Verhaak RGW, Tamboli P, Torres-Garcia W, Akbani R, Weinstein JN, Reuter V, Hsieh JJ, Brannon AR, Hakimi AA, Jacobsen A, Ciriello G, Reva B, Ricketts CJ, Linehan WM, Stuart JM, Rathmell WK, Shen H, Laird PW, Muzny D, Davis C, Xi L, Chang K, Kakkar N, Treviño LR, Benton S, Reid JG, Morton D, Doddapaneni H, Han Y, Lewis L, Dinh H, Kovar C, Zhu Y, Santibanez J, Wang M, Hale W, Kalra D, Getz G, Lawrence MS, Sougnez C, Carter SL, Sivachenko A, Lichtenstein L, Stewart C, Voet D, Fisher S, Gabriel SB, Lander E, Schumacher SE, Tabak B, Saksena G, Onofrio RC, Cherniack AD, Gentry J, Ardlie K, Meyerson M, Chun HJE, Mungall AJ, Sipahimalani P, Stoll D, Ally A, Balasundaram M, Butterfield YSN, Carlsen R, Carter C, Chuah E, Coope RJN, Dhalla N, Gorski S, Guin R, Hirst C, Hirst M, Holt RA, Lebovitz C, Lee D, Li HYI, Mayo M, Moore RA, Pleasance E, Plettner P, Schein JE, Shafiei A, Slobodan JR, Tam A, Thiessen N, Varhol RJ, Wye N, Zhao Y, Birol I, Jones SJM, Marra MA, Auman JT, Tan D, Jones CD, Hoadley KA, Mieczkowski PA, Mose LE, Jefferys SR, Topal MD, Liquori C, Turman YJ, Shi Y, Waring S, Buda E, Walsh J, Wu J, Bodenheimer T, Hoyle AP, Simons JV, Soloway MG, Balu S, Parker JS, Hayes DN, Perou CM, Kucherlapati R, Park P, Triche T, Weisenberger DJ, Lai PH, Bootwalla MS, Maglinte DT, Mahurkar S, Berman BP, Berg DJ Van Den, Cope L, Baylin SB, Noble MS, DiCara D, Zhang H, Cho J, Heiman DI, Gehlenborg N, Mallard W, Lin P, Frazer S, Stojanov P, Liu Y, Zhou L, Kim J, Chin L, Benz C, Yau C, Reynolds SM, Shmulevich I, Vegesna R, Kim H, Zhang W, Cogdell D, Jonasch E, Ding Z, Lu Y, Zhang N, Unruh AK, Casasent TD, Wakefield C, Tsavachidou D, Mills GB, Gao J, Cerami E, Gross B, Aksoy BA, Sinha R, Weinhold N, Sumer SO, Taylor BS, Shen R, Ostrovnaya I, Berger MF, Ladanyi M, Sander C, Fei SS, Stout A, Spellman PT, Rubin DL, Liu TT, Ng S, Paull EO, Carlin D, Goldstein T, Waltman P, Ellrott K, Zhu J, Haussler D, Xiao W, Shelton C, Gardner J, Penny R, Sherman M, Mallery D, Morris S, Paulauskis J, Burnett K, Shelton T, Kaelin WG, Choueiri T, Atkins MB, Curley E, Tickoo S, Thorne L, Boice L, Huang M, Fisher JC, Vocke CD, Peterson J, Worrell R, Merino MJ, Schmidt LS, Czerniak BA, Aldape KD, Wood CG, Boyd J, Weaver JE, Iacocca MV, Petrelli N, Witkin G, Brown J, Czerwinski C, Huelsenbeck-Dill L, Rabeno B, Myers J, Morrison C, Bergsten J, Eckman J, Harr J, Smith C, Tucker K, Zach LA, Bshara W, Gaudioso C, Dhir R, Maranchie J, Nelson J, Parwani A, Potapova CO, Fedosenko K, Cheville JC, Thompson RH, Mosquera JM, Rubin MA, Blute ML, Pihl T, Jensen M, Sfeir R, Kahn A, Chu A, Kothiyal P, Snyder E, Pontius J, Ayala B, Backus M, Walton J, Baboud J, Berton D, Nicholls M, Srinivasan D, Raman R, Girshik S, Kigonya P, Alonso S, Sanbhadti R, Barletta S, Pot D, Sheth M, Demchok JA, Davidsen T, Wang Z, Yang L, Tarnuzzer RW, Zhang J, Eley G, Ferguson ML, Mills Shaw KR, Guyer MS, Ozenberger BA, Sofia HJ. Comprehensivemolecular characterization of clear cell renal cell carcinoma. *Nature* 2013;**499**:43–49.

8. Levey AS, Stevens LA, Schmid CH, Zhang Y, Castro AF, Feldman HI, Kusek JW, Eggers P, Lente F Van, Greene T, Coresh J. A new equation to estimate glomerular filtration rate. *Ann Intern Med* 2009;**150**:604–612.

9. Purcell S, Neale B, Todd-Brown K, Thomas L, Ferreira MAR, Bender D, Maller J, Sklar P, Bakker PIW De, Daly MJ, Sham PC. PLINK: A tool set for whole-genome association and population-based linkage analyses. *Am J Hum Genet* 2007;**81**:559–575.

10. Andrews S. FastQC - A quality control tool for high throughput sequence data. 2010. http://www.bioinformatics.babraham.ac.uk/projects/fastqc

11. Bray NL, Pimentel H, Melsted P, Pachter L. Near-optimal probabilistic RNA-seq quantification. *Nat Biotechnol* 2016;**34**:525–527.

12. Young MD, Mitchell TJ, Vieira Braga FA, Tran MGB, Stewart BJ, Ferdinand JR, Collord G, Botting RA, Popescu DM, Loudon KW, Vento-Tormo R, Stephenson E, Cagan A, Farndon SJ, Velasco-Herrera MDC, Guzzo C, Richoz N, Mamanova L, Aho T, Armitage JN, Riddick ACP, Mushtaq I, Farrell S, Rampling D, Nicholson J, Filby A, Burge J, Lisgo S, Maxwell PH, Lindsay S, Warren AY, Stewart GD, Sebire N, Coleman N, Haniffa M, Teichmann SA, Clatworthy M, Behjati S. Single-cell transcriptomes from human kidneys reveal the cellular identity of renal tumors. *Science* 2018;**361**:594–599.

13. Butler A, Hoffman P, Smibert P, Papalexi E, Satija R. Integrating single-cell transcriptomic data across different conditions, technologies, and species. *Nat Biotechnol* 2018;**36**:411–420.

14. Tukiainen T, Villani AC, Yen A, Rivas MA, Marshall JL, Satija R, Aguirre M, Gauthier L, Fleharty M, Kirby A, Cummings BB, Castel SE, Karczewski KJ, Aguet F, Byrnes A, Gelfand ET, Getz G, Hadley K, Handsaker RE, Huang KH, Kashin S, Lek M, Li X, Nedzel JL, Nguyen DT, Noble MS, Segrè AV, Trowbridge CA, Abell NS, Balliu B, Barshir R, Basha O, Battle A, Bogu GK, Brown A, Brown CD, Chen LS, Chiang C, Conrad DF, Cox NJ, Damani FN, Davis JR, Delaneau O, Dermitzakis ET, Engelhardt BE, Eskin E, Ferreira PG, Frésard L, Gamazon ER, Garrido-Martín D, Gewirtz ADH, Gliner G, Gloudemans MJ, Guigo R, Hall IM, Han B, He Y, Hormozdiari F, Howald C, Im HK, Jo B, Kang EY, Kim Y, Kim-Hellmuth S, Lappalainen T, Li G, Li X, Liu B, Mangul S, McCarthy MI, McDowell IC, Mohammadi P, Monlong J, Montgomery SB, Muñoz-Aguirre M, Ndungu AW, Nicolae DL, Nobel AB, Oliva M, Ongen H, Palowitch JJ, Panousis N, Papasaikas P, Park Y, Parsana P, Payne AJ, Peterson CB, Quan J, Reverter F, Sabatti C, Saha A, Sammeth M, Scott AJ, Shabalin AA, Sodaei R, Stephens M, Stranger BE, Strober BJ, Sul JH, Tsang EK, Urbut S, Bunt M Van De, Wang G, Wen X, Wright FA, Xi HS, Yeger-Lotem E, Zappala Z, Zaugg JB, Zhou YH, Akey JM, Bates D, Chan J, Claussnitzer M, Demanelis K, Diegel M, Doherty JA, Feinberg AP, Fernando MS, Halow J, Hansen KD, Haugen E, Hickey PF, Hou L, Jasmine F, Jian R, Jiang L, Johnson A, Kaul R, Kellis M, Kibriya MG, Lee K, Li JB, Li Q, Lin J, Lin S, Linder S, Linke C, Liu Y, Maurano MT, Molinie B, Nelson J, Neri FJ, Park Y, Pierce BL, Rinaldi NJ, Rizzardi LF, Sandstrom R, Skol A, Smith KS, Snyder MP, Stamatoyannopoulos J, Tang H, Wang L, Wang M, Wittenberghe N Van, Wu F, Zhang R, Nierras CR, Branton PA, Carithers LJ, Guan P, Moore HM, Rao A, Vaught JB, Gould SE, Lockart NC, Martin C, Struewing JP, Volpi S, Addington AM, Koester SE, Little AR, Brigham LE, Hasz R, Hunter M, Johns C, Johnson M, Kopen G, Leinweber WF, Lonsdale JT, McDonald A, Mestichelli B, Myer K, Roe B, Salvatore M, Shad S, Thomas JA, Walters G, Washington M, Wheeler J, Bridge J, Foster BA, Gillard BM, Karasik E, Kumar R, Miklos M, Moser MT, Jewell SD, Montroy RG, Rohrer DC, Valley DR, Davis DA, Mash DC, Undale AH, Smith AM, Tabor DE, Roche N V., McLean JA, Vatanian N, Robinson KL, Sobin L, Barcus ME, Valentino KM, Qi L, Hunter S, Hariharan P, Singh S, Um KS, Matose T, Tomaszewski MM, Barker LK, Mosavel M, Siminoff LA, Traino HM, Flicek P, Juettemann T, Ruffier M, Sheppard D, Taylor K, Trevanion SJ, Zerbino DR, Craft B, Goldman M, Haeussler M, Kent WJ, Lee CM, Paten B, Rosenbloom KR, Vivian J, Zhu J, Regev A, Ardlie KG, Hacohen N, MacArthur DG. Landscape of X chromosome inactivation across human tissues. *Nature* 2017;**550**:244–248.

15. Mi H, Muruganujan A, Ebert D, Huang X, Thomas PD. PANTHER version 14: More genomes, a new PANTHER GO-slim and improvements in enrichment analysis tools. *Nucleic Acids Res* 2019;**47**:D419–D426.

16. Leek JT, Johnson WE, Parker HS, Jaffe AE, Storey JD. The SVA package for removing batch effects and other unwanted variation in high-throughput experiments. *Bioinformatics* 2012;**28**:882–883.

17. Leek JT, Storey JD. Capturing heterogeneity in gene expression studies by surrogate variable analysis. *PLoS Genet* 2007;**3**:1724–1735.

18. Joubert BR, Felix JF, Yousefi P, Bakulski KM, Just AC, Breton C, Reese SE, Markunas CA, Richmond RC, Xu CJ, Küpers LK, Oh SS, Hoyo C, Gruzieva O, Söderhäll C, Salas LA, Baïz N, Zhang H, Lepeule J, Ruiz C, Ligthart S, Wang T, Taylor JA, Duijts L, Sharp GC, Jankipersadsing SA, Nilsen RM, Vaez A, Fallin MD, Hu D, Litonjua AA, Fuemmeler BF, Huen K, Kere J, Kull I, Munthe-Kaas MC, Gehring U, Bustamante M, Saurel-Coubizolles MJ, Quraishi BM, Ren J, Tost J, Gonzalez JR, Peters MJ, Håberg SE, Xu Z, Meurs JB Van, Gaunt TR, Kerkhof M, Corpeleijn E, Feinberg AP, Eng C, Baccarelli AA, Benjamin Neelon SE, Bradman A, Merid SK, Bergström A, Herceg Z, Hernandez-Vargas H, Brunekreef B, Pinart M, Heude B, Ewart S, Yao J, Lemonnier N, Franco OH, Wu MC, Hofman A, McArdle W, Vlies P Van Der, Falahi F, Gillman MW, Barcellos LF, Kumar A, Wickman M, Guerra S, Charles MA, Holloway J, Auffray C, Tiemeier HW, Smith GD, Postma D, Hivert MF, Eskenazi B, Vrijheid M, Arshad H, Antó JM, Dehghan A, Karmaus W, Annesi-Maesano I, Sunyer J, Ghantous A, Pershagen G, Holland N, Murphy SK, Demeo DL, Burchard EG, Ladd-Acosta C, Snieder H, Nystad W, Koppelman GH, Relton CL, Jaddoe VWV, Wilcox A, Melén E, London SJ. DNA Methylation in Newborns and Maternal Smoking in Pregnancy: Genome-wide Consortium Meta-analysis. *Am J Hum Genet* 2016;**98**:680–696.

19. Borenstein M, Hedges LV, Higgins JPT, Rothstein HR. Introduction to Meta-Analysis. Introd. to Meta-Analysis. 2009.

20. Storey JD, Tibshirani R. Statistical significance for genomewide studies. *Proc Natl Acad Sci U S A* 2003;**100**:9440–9445.

21. Storey JD, Bass AJ, Dabney A, Robinson D. qvalue: Q-value estimation for false discovery rate control. R package version 2.18.0. 2019. http://www.bioconductor.org/packages/release/bioc/html/qvalue.html

22. Gamazon ER, Segrè AV, Bunt M Van De, Wen X, Xi HS, Hormozdiari F, Ongen H, Konkashbaev A, Derks EM, Aguet F, Quan J, Nicolae DL, Eskin E, Kellis M, Getz G, McCarthy MI, Dermitzakis ET, Cox NJ, Ardlie KG. Using an atlas of gene regulation across 44 human tissues to inform complex disease- and trait-associated variation. *Nat Genet* 2018;**50**:956–967.

23. Ritchie ME, Phipson B, Wu D, Hu Y, Law CW, Shi W, Smyth GK. Limma powers differential expression analyses for RNA-sequencing and microarray studies. *Nucleic Acids Res* 2015;**43**:e47.

24. Ju W, Nair V, Smith S, Zhu L, Shedden K, Song PXK, Mariani LH, Eichinger FH, Berthier CC, Randolph A, Lai JYC, Zhou Y, Hawkins JJ, Bitzer M, Sampson MG, Their M, Solier C, Duran-Pacheco GC, Duchateau-Nguyen G, Essioux L, Schott B, Formentini I, Magnone MC, Bobadilla M, Cohen CD, Bagnasco SM, Barisoni L, Lv J, Zhang H, Wang HY, Brosius FC, Gadegbeku CA, Kretzler M. Tissue transcriptome-driven identification of epidermal growth factor as a chronic kidney disease biomarker. *Sci Transl Med* 2015;**7**:7071.

25. Sampson MG, Robertson CC, Martini S, Mariani LH, Lemley KV, Gillies CE, Otto EA, Kopp JB, Randolph A, Vega-Warner V, Eichinger F, Nair V, Gipson DS, Cattran DC, Johnstone DB, O’Toole JF, Bagnasco SM, Song PX, Barisoni L, Troost JP, Kretzler M, Sedor JR. Integrative genomics identifies novel associations with apol1 risk genotypes in black neptune subjects. *J Am Soc Nephrol* 2016;**27**:814–823.

26. Woroniecka KI, Park ASD, Mohtat D, Thomas DB, Pullman JM, Susztak K. Transcriptome analysis of human diabetic kidney disease. *Diabetes* 2011;**60**:2354–2369.

27. Beckerman P, Qiu C, Park J, Ledo N, Ko YA, Park ASD, Han SY, Choi P, Palmer M, Susztak K. Human Kidney Tubule-Specific Gene Expression Based Dissection of Chronic Kidney Disease Traits. *EBioMedicine* 2017;**24**:267–276.
